# ABCC10-mediated cGAMP efflux drives cancer cell radiotherapy resistance

**DOI:** 10.1038/s41418-025-01552-1

**Published:** 2025-08-06

**Authors:** Zhengyang Zhang, Jie Gao, Xiang Liao, Zining Zhang, Xiongfeng Cao, Yi Gong, Wenlong Chen, Lirong Zhang, Hsiang-i Tsai, Dongqing Wang, Haitao Zhu

**Affiliations:** 1 Institute of Medical Imaging and Artificial Intelligence, Jiangsu University, Zhenjiang, China; 2 Department of Medical Imaging, The Affiliated Hospital of Jiangsu University, Zhenjiang, China

**Keywords:** Cancer metabolism, Oncogenes

## Abstract

Although radiotherapy (RT) is used in more than 50% of cancer patients, the intrinsic radioresistance of cancer cells, characterized by metabolic adaptation, significantly limits its clinical efficacy. However, the mechanisms underlying RT resistance (RTR) remain incompletely understood. In this study, we used high-throughput metabolic CRISPR library screening and identified ABCC10 as a novel molecular contributor to RTR. Functional assays, including vesicle transport, molecular docking, and an enzyme-linked immunosorbent assay, confirmed that the R545 site of ABCC10 binds to and effluxes 2′3′-cyclic GMP–AMP (cGAMP) in an ATP-dependent manner. Mechanistically, RNA transcriptomics, along with overexpression and silencing experiments, demonstrated that ABCC10-mediated export of cGAMP suppresses the STING-TBK1-IRF3 signaling pathway. This efflux reduces RT-induced intercellular accumulation of reactive oxygen species and DNA damage. In vivo, a combination of RT and nilotinib, a potential ABCC10 inhibitor, synergistically inhibited tumor growth. In summary, we identified ABCC10 as a novel exporter of cGAMP in RTR. Our results highlight its potential role as a biomarker for predicting RT response and as a therapeutic target for overcoming RTR.

## Introduction

More than 50% of cancer patients require curative, palliative, or adjuvant radiotherapy (RT) [1]. Despite its widespread use, RT resistance (RTR) remains a significant challenge, and paradoxically, can even accelerate tumor progression [2]. This resistance is closely linked to the heterogeneous response of cancer cells and the surrounding tumor microenvironment to ionizing radiation [3, 4]. However, the precise mechanisms driving RTR are not fully understood and warrant further in-depth investigation.

In response to RT-induced DNA damage, cancer cells can develop various resistance mechanisms, including enhanced DNA repair capabilities and tumor microenvironment remodeling. In addition, metabolic reprogramming—a hallmark of malignancy—enables cancer cells to survive during RT [5, 6]. Cancer cells increase the metabolic flux of glucose, amino acids, fatty acids, and other substrates, providing the necessary resources for the scavenging of reactive oxygen species (ROS) and DNA repair [7]. For instance, glutaminase-mediated biosynthesis of glutamate facilitates nucleotide synthesis and glutathione production, which are crucial for DNA repair and the prevention of oxidative stress, contributing to RTR in IDH-mutant glioma cells [8]. Therefore, targeting metabolic reprogramming represents a promising therapeutic strategy for enhancing radiosensitivity. In addition to the classical metabolic pathways involving glucose, amino acids, and fatty acids, we investigated whether other metabolic fluxes, such as nucleotides, play an essential role in driving RTR.

RT-induced DNA damage activates the cyclic GMP-AMP synthase (cGAS)-stimulator of interferon genes (STING) pathway. In addition to its role as a downstream effector of DNA damage, STING activation has also been implicated in the regulation and maintenance of DNA damage response pathways. Nassour et al. [9] demonstrated that STING activation, triggered by cytoplasmic DNA from telomere crisis-induced chromosomal instability, induces excessive autophagy which executes cell death. This process is crucial for eliminating genomically unstable pre-cancerous cells and acts as a potent tumor-suppressive mechanism. Similarly, Ranoa et al. [10] demonstrated that STING deficiency compromises DNA damage surveillance, leading to aberrant mitotic entry and accumulated chromosomal instability, which underlies its tumor-suppressive function in early carcinogenesis. However, in the context of radiotherapy, STING signaling exhibits dual roles in antitumor immunity. For instance, in dendritic cells within irradiated tumors, cGAS-STING activation promotes both canonical NF-κB-mediated type I interferon production and non-canonical NF-κB signaling, which antagonizes interferon expression by inhibiting RelA activity [11]. This immunosuppressive axis significantly limits the efficacy of radiotherapy. Therefore, STING activation plays diverse, and at times opposing, roles in the context of RT efficacy. However, the precise checkpoints that determine whether STING has tumor-suppressive or tumor-promoting functions remain unclear.

2′3′-cyclic GMP–AMP (cGAMP) is a key molecule in the cGAS-STING pathway [12]. Under conditions of cellular stress, cGAS-activated cGAMP rapidly diffuses throughout the cell, acting as a second messenger to induce STING translocation and activation. However, recent studies have challenged this conventional view, revealing that cGAMP can be exported by cancer cells in a paracrine manner, activating STING in neighboring cells independent of cGAS [13, 14]. Notably, X-ray irradiation significantly enhances the capacity of cancer cells to export cGAMP [15]. Secreted cGAMP is subsequently taken up by non-cancerous cells, where it amplifies antitumor immunity in a STING-dependent manner [16, 17]. However, the overall impact of cGAMP export on the intrinsic STING responses of cancer cells and its effect on therapy outcomes remain unclear. Moreover, as cGAMP cannot passively cross cellular membranes, the crucial transporter responsible for cGAMP export under various therapeutic stressors remains incompletely understood and warrants further investigation.

In this study, using high-throughput metabolic CRISPR library screening, we identified ATP Binding Cassette Subfamily C Member 10 (ABCC10), a plasma membrane-localized transporter, as a novel mediator of RTR. For the first time, we demonstrated that the R545 site of ABCC10 binds to and exports cGAMP in an ATP-dependent manner. Furthermore, ABCC10-mediated cGAMP export limits the activation of the STING-TBK1-IRF3 pathway, independent of cGAS, following RT. This ultimately reduces RT-induced intercellular ROS accumulation and DNA damage. Our findings suggest that ABCC10, a novel cGAMP transporter, drives RTR in cancer cells, highlighting its potential usefulness as a biomarker for predicting RT response and as a target for therapeutic strategies to overcome resistance.

## Results

### ABCC10 is involved in RTR

**Fig. 1:**
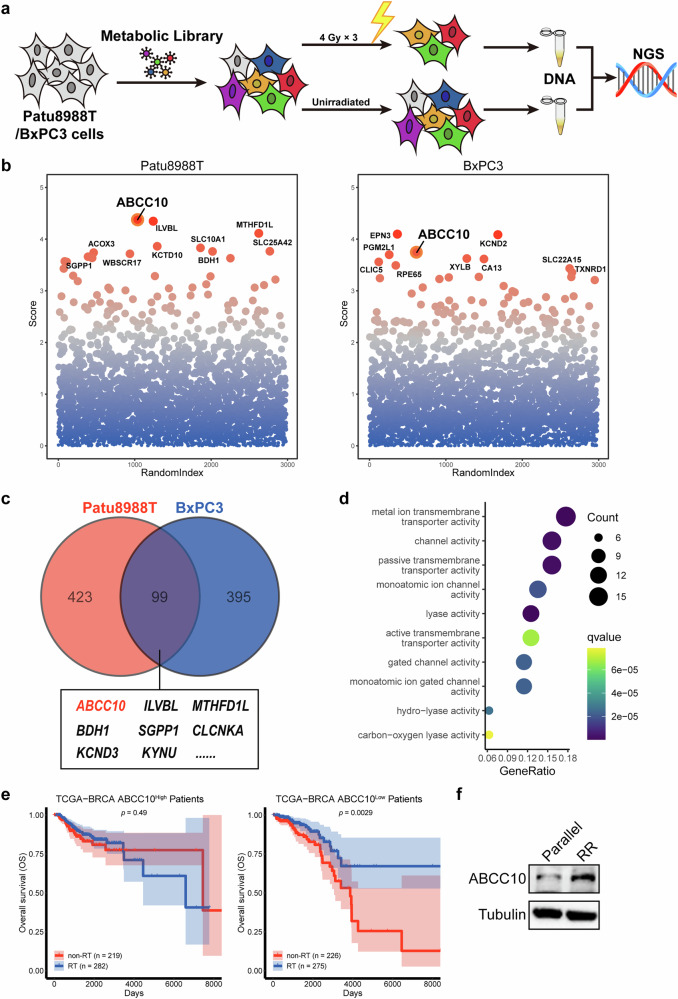
Human metabolic CRISPR-Cas9 screen to identify novel candidates for radiotherapy resistance. **a** Schematic of a metabolic in vitro CRISPR screen for identifying regulators of RT. **b** Scatter plot showing the top 10 negative regulatory genes in two rounds of the CRISPR-Cas9 screen using MAGeCK analysis. **c** Indication of the combined analysis of the two rounds of the functional screen. **d** Gene ontology (GO) analysis of molecular function (MF) for the intersection of 99 negative regulatory genes. **e** Survival analysis for BRCA patients was conducted separately within the high and low expression groups, comparing patients treated with RT and those who did not receive RT. **f** Immunoblot analysis of the protein expression levels of ABCC10 in parallel Patu8988T cells and radioresistant Patu8988T cells.

To systematically identify crucial, previously unrecognized, metabolites involved in the effects of RT, a human metabolic CRISPR-Cas9 screening initiative was conducted. We screened approximately 30,000 single-guide RNAs (sgRNAs) targeting around 3,000 human metabolic genes. Two classical RT-resistant human pancreatic cancer cell lines, Patu8988T and BxPC3, were selected for the screening [18]. Based on previous studies and preliminary experiments [19], three radiation doses of 4 Gy were selected as it reduced cell viability by 50% and increased the sensitivity of genetic screens (Supplementary Fig. 1a). To minimize the impact of these sgRNAs on cell viability, the abundance of each residual sgRNA in treated cancer cells was compared to that of parallel control cells using next-generation sequencing (Fig. 1a). MAGeCK analysis identified 522 candidate metabolic genes in Patu8988T and 494 in BxPC3 in response to irradiation (Fig. 1b). Gene enrichment analysis revealed that the common RTR-associated candidate genes in both cell lines were involved in similar metabolic pathways, such as purine metabolism and the PPAR signaling pathway (Supplementary Fig. 1b–d), consistent with previously published RTR mechanisms [20, 21]. The top five genes identified from the screening were consistently overexpressed in cancer tissues, further demonstrating the reliability of the high-throughput metabolic CRISPR library screening (Supplementary Fig. 1e, f).

Among the screening hits, ABCC10 consistently ranked prominently, placing first in the Patu8988T screen and third in the BxPC3 screen (Fig. 1b, c). As a member of the ATP-binding cassette (ABC) transporter family, ABCC10 causes extracellular efflux of materials. The identification of ABCC10 in RTR aligns with the Gene Ontology (GO) molecular function enrichment analysis, which highlights the crucial role of transmembrane transporters in modulating radiation sensitivity (Fig. 1d). To further investigate the relationship between ABCC10 and RT outcomes, we analyzed survival data from The Cancer Genome Atlas (TCGA) for breast cancer, head, and neck cancer, and pancreatic cancer. Patients with low ABCC10 expression had longer survival times following RT compared to those with high ABCC10 expression (Fig. 1e and Supplementary Fig. 2a, b). In addition, the expression levels of ABCC10 were significantly higher in radioresistant cell lines compared to their corresponding control cancer cells, suggesting that ABCC10 is a crucial determinant of RTR (Fig. 1f and Supplementary Fig. 2c, d).

### ABCC10 is implicated in cancer cell RTR

**Fig. 2:**
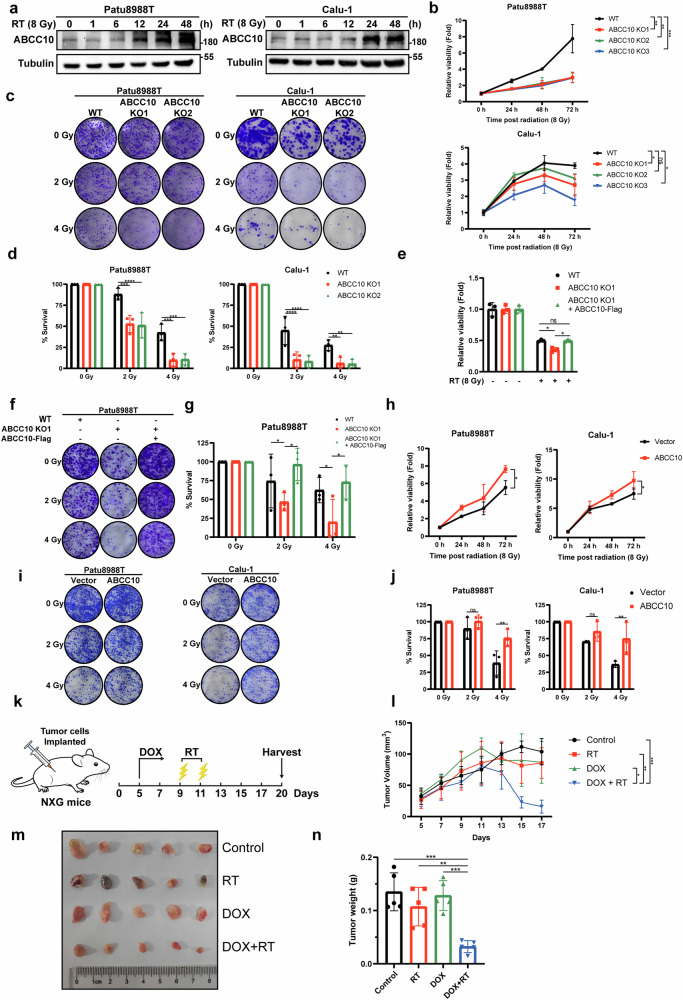
ABCC10 confers radioresistance to neoplastic cells. **a** Immunoblot analysis of the protein expression levels of ABCC10 in Patu8988T and Calu-1 cells treated with radiotherapy (RT) (8 Gy) at the indicated time points. **b** Cell viability in Patu8988T and Calu-1 ABCC10-knockout cells treated with RT (8 Gy). Representative images (**c**) and quantification (**d**) of clonogenic survival analysis of Patu8988T and Calu-1 ABCC10-knockout cells treated with the indicated dose of ionizing radiation. **e** Cell viability in WT cells, ABCC10-knockout cells and ABCC10-knockout cells with re-expression of ABCC10 treated with RT (8 Gy). Representative images (**f**) and quantification (**g**) of clonogenic survival analysis of WT cells, ABCC10-knockout cells and ABCC10-knockout cells with re-expression of ABCC10 treated with the indicated dose of ionizing radiation. **h** Cell viability in Patu8988T and Calu-1 ABCC10-overexpressing cells treated with RT (8 Gy). Representative images (**i**) and quantification (**j**) of clonogenic survival analysis of Patu8988T and Calu-1 ABCC10-overexpressing cells treated with the indicated dose of ionizing radiation. **k–n** NXG mice were transplanted subcutaneously with doxycycline (DOX)-inducible ABCC10-knockdown Patu8988T cells and treated as indicated. A diagram of tumor growth delay experiments performed in vivo and the ionizing radiation fractionated treatment protocol is shown in (**k**); tumor volumes were calculated (**l**); tumor images were acquired as shown in (**m**); tumor weights (**n**) were measured. Experiments were repeated three times, and data are expressed as mean ± SEM (*p < 0.05, **p < 0.01, ***p < 0.001, ****p < 0.0001).

To elucidate the role of ABCC10 in RTR, its expression levels were assessed in a panel of RT-resistant cancer cell lines, including human pancreatic cancer cells (Patu8988T and BxPC3), human non-small cell lung cancer cells (Calu-1), and human breast cancer cells (MCF-7). Consistent upregulation of ABCC10 following RT was observed in a time-dependent manner (Fig. 2a and Supplementary Fig. 3a). To further explore the relationship between ABCC10 and RTR, ABCC10-knockout cells were generated using CRISPR-Cas9 technology, and knockdown cells were established using doxycycline (DOX)-inducible short hairpin RNA (shRNA) targeting ABCC10. The high efficiency of ABCC10 silencing was confirmed by Western blotting and qPCR analysis (Supplementary Fig. 3b–d). As expected, compared to their respective control parental cancer cells, the viability of both ABCC10-knockout and ABCC10-knockdown cells was significantly reduced in response to RT (Fig. 2b and Supplementary Fig. 3e, f). Similarly, clonogenic survival assays revealed that ABCC10-knockout or ABCC10-knockdown cells exhibited a significant reduction in colony formation following 2 or 4 Gy irradiation (Fig. 2c, d and Supplementary Fig. 3g–i).

To further validate that the observed phenotypes were specifically due to ABCC10 loss, we performed a rescue experiment. A plasmid encoding ABCC10 with a C-terminal FLAG tag and a different antibiotic selection marker was introduced into ABCC10-knockout cells to generate a stable complemented line. Remarkably, re-expression of ABCC10 restored the radioresistant phenotype of the KO cells, as evidenced by increased cell viability (Fig. 2e), enhanced clonogenic survival (Fig. 2f, g), and reversal of the sensitized phenotype observed in ABCC10-deficient cells. These results confirm the functional requirement of ABCC10 in mediating resistance to RT.

To further investigate the functional role of ABCC10 in RTR, a plasmid expressing ABCC10 fused with a FLAG tag was constructed. Following the selection of transduced cells, discernible FLAG protein expression was confirmed (Supplementary Fig. 3j). Notably, ABCC10-overexpressing cancer cells exhibited significantly increased cell viability and enhanced colony formation ability under various doses of irradiation (Fig. 2h–j). Similar results were observed in ABCC10-overexpressing cancer cells generated using CRISPR activation technology (Supplementary Fig. 3k–m).

To further validate our in vitro findings on ABCC10-mediated RTR, NXG mice (n = 5 per group) bearing 1 × 10^7^ Patu8988T cells, transduced with DOX-inducible shRNA targeting ABCC10, were randomly divided into the following groups: control, RT, DOX, and DOX + RT (Fig. 2k). Compared to controls, RT treatment moderately reduced tumor volume and weight, but this effect was significantly enhanced in the presence of DOX. This suggests that DOX-induced ABCC10 knockdown effectively reversed RTR in the cancer cells (Fig. 2l–n). Overall, these findings indicate that the expression levels of ABCC10 play a critical role in determining RT sensitivity.

### ABCC10 alleviates ROS-dependent DNA damage, enhancing RTR

**Fig. 3:**
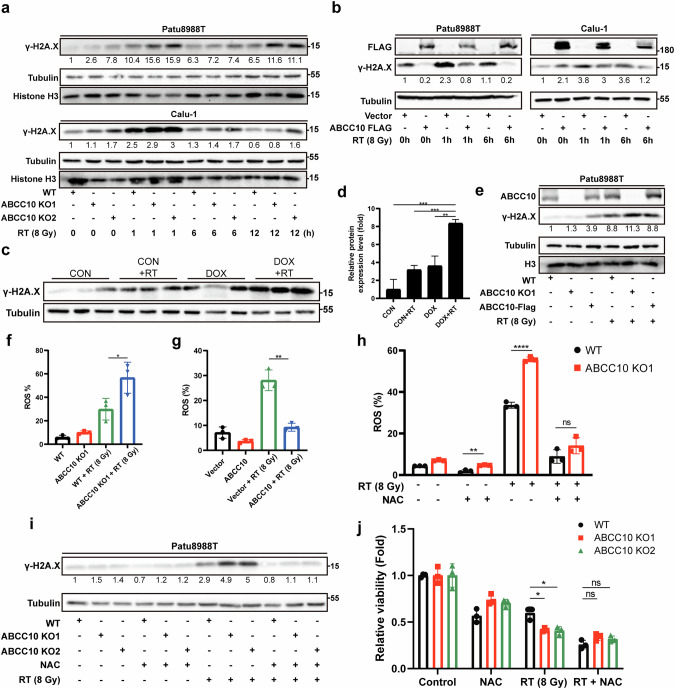
ABCC10 alleviates post-radiotherapy ROS-dependent DNA damage. **a** Immunoblot analysis of the protein expression levels of γ-H2A.X in Patu8988T and Calu-1 ABCC10-knockout cells at the indicated times after RT (8 Gy). **b** Immunoblot analysis of the protein expression levels of γ-H2A.X in Patu8988T and Calu-1 ABCC10-overexpressing cells at the indicated times after RT. **c** Protein levels of γ-H2A.X in tumor tissues were detected. **d** Quantification of γ-H2A.X protein levels in in tumor tissues. **e** Immunoblot analysis of the protein expression levels of ABCC10 and γ-H2A.X in WT cells, ABCC10-knockout cells and ABCC10-knockout cells with re-expression of ABCC10 treated with RT (8 Gy) after 6 h. **f** Quantification of flow cytometry-based analysis of ROS levels in Patu8988T WT and ABCC10 KO cells at 24 h after RT. **g** Quantification of flow cytometry-based analysis of ROS levels in Patu8988T vector and ABCC10-overexpressing cells at 24 h after RT. **h** Quantification of flow cytometry-based analysis of ROS levels in WT and ABCC10 KO1 Patu8988T cells pretreated with or without NAC (1 mM) at 24 h after RT. **i** Immunoblot analysis of the protein expression levels of γ-H2A.X in WT and ABCC10 KO1 Patu8988T cells pretreated with or without NAC (1 mM) at 6 h after RT. **j** Cell viability of Patu8988T WT and ABCC10-knockout cells pretreated with or without NAC (1 mM) at 72 h after RT. Experiments were repeated three times, and the data are expressed as mean ± SEM (*p < 0.05, **p < 0.01, ***p < 0.001, ****p < 0.0001).

DNA damage is the primary cause of RT-induced cell death, and we sought to determine whether ABCC10 plays a role in regulating this process. Interestingly, compared to control cells, the expression of γ-H2A.X, a biomarker of DNA damage, was upregulated with a peak at 1 h post-RT. In ABCC10-knockout cells, γ-H2A.X levels were further elevated, indicating sustained DNA damage (Fig. 3a and Supplementary Fig. 4a). Conversely, ABCC10 overexpression resulted in a reversed pattern, with reduced γ-H2A.X expression following RT (Fig. 3b and Supplementary Fig. 4b). Moreover, we also observed elevated γ-H2A.X levels in the DOX + RT group with ABCC10 knockdown from the protein profiling of tumor tissues, indicating more pronounced DNA damage (Fig. 3c, d). Consistently, comet assays revealed a significant increase in tail moment in ABCC10-knockout cells post-irradiation, further indicating augmented DNA damage (Supplementary Fig. 4c, d). In addition, re-expression of ABCC10 in knockout cells significantly attenuated RT-induced γ-H2A.X expression (Fig. 3e), providing further evidence that the increased DNA damage in ABCC10-deficient cells is directly attributable to the loss of ABCC10 function. These findings demonstrate that ABCC10 modulates the severity of RT-induced DNA damage.

RT induces DNA damage through both ROS-dependent and ROS-independent mechanisms. We observed a marked increase in intracellular ROS in ABCC10-knockdown cancer cells, whereas ABCC10 overexpression resulted in insufficient ROS generation (Fig. 3f, g and Supplementary Fig. 4e, f). To further investigate the relationships between ABCC10, ROS levels, and DNA damage, ABCC10-knockout cancer cells were or were not pretreated with N-acetyl cysteine (NAC), a classical ROS scavenger, following RT. Notably, NAC effectively reduced the elevated intracellular ROS levels, decreased γ-H2A.X expression, and restored cell viability in RT-treated ABCC10-knockout cells. These findings indicate that intracellular ROS serves as a key link between ABCC10 and DNA damage (Fig. 3h–j and Supplementary Fig. 4g). Collectively, these results suggest that ABCC10 mitigates intracellular ROS and DNA damage, thereby contributing to RTR.

### Role of the STING pathway in ABCC10-mediated alleviation of ROS production and DNA damage

**Fig. 4:**
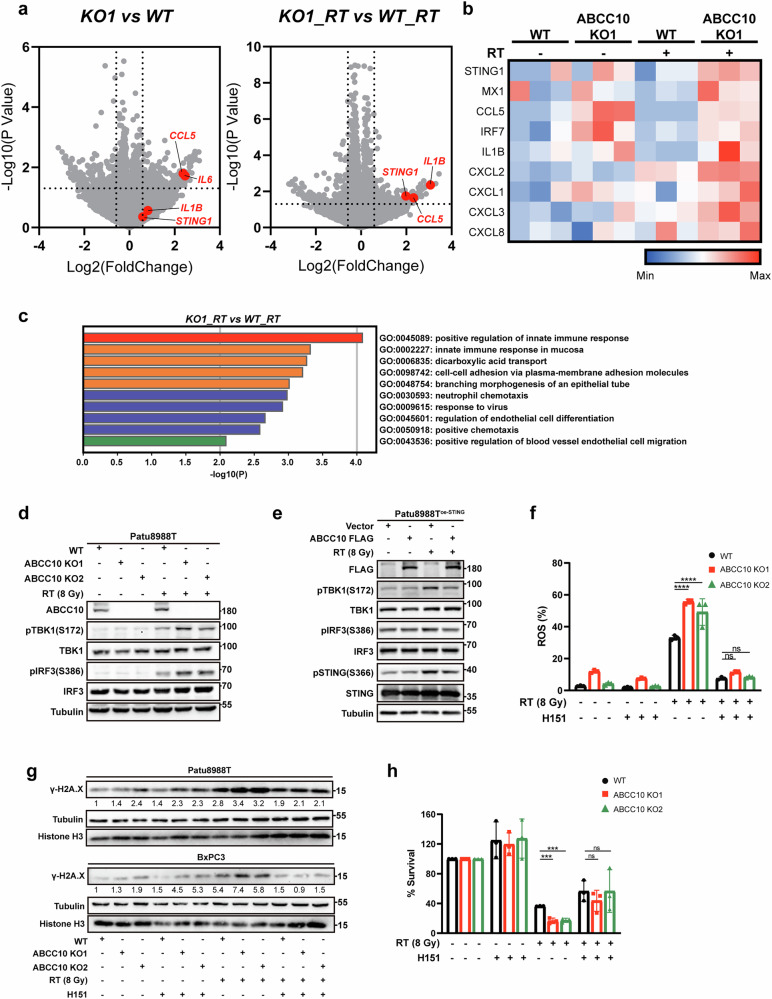
ABCC10 loss further activates the STING pathway after radiation. **a** A volcanic map was used to analyze differentially expressed transcriptome genes of ABCC10 KO1 cells treated with or without radiotherapy (RT) for 6 h. **b** Heatmap of STING pathway-related genes in ABCC10 KO1 cells compared to WT cells. **c** Gene Ontology (GO) molecular function enrichment analysis of upregulated genes from ABCC10 KO1 Patu8988T cells compared to WT cells treated with RT. **d** Western blot analysis of ABCC10, pTBK1, TBK1, pIRF3, and IRF3 protein levels in ABCC10 WT and KO Patu8988T cells. **e** Western blot analysis of FLAG, pTBK1, TBK1, pIRF3, IRF3, pSTING, and STING protein levels in vector and ABCC10-overexpressing Patu8988T STING-overexpression (OE) cells. **f** Quantification of flow cytometry-based analysis of reactive oxygen species (ROS) levels in Patu8988T WT and ABCC10 KO cells pretreated with H151 24 h after RT. **g** Western blot analysis of γ-H2A.X protein levels in Patu8988T and BxPC3 WT and ABCC10 KO cells pretreated with H151 (1 μM) 6 h after RT. **h** Quantification of clonogenic survival analysis of Patu8988T WT and ABCC10 KO cells pretreated with H151 (1 μM) after RT. Experiments were repeated at least three times, and the data are expressed as mean ± SEM (*p < 0.05, **p < 0.01, ***p < 0.001, ****p < 0.0001).

To elucidate the underlying mechanisms by which ABCC10 alleviates intracellular ROS levels and DNA damage following RT, RNA transcriptomic sequencing was performed on ABCC10-knockout and wild-type Patu8988T cells. In all, 351 and 387 differentially expressed genes were identified with and without RT, respectively (Fig. 4a). Among these, genes associated with the STING pathway were significantly upregulated in ABCC10-knockout cells, with further enhancement observed following RT (Fig. 4a, b). Activated STING facilitates the recruitment and phosphorylation of TBK1 and IRF3, which subsequently translocate to the nucleus, triggering the expression of various interferon-stimulated genes (ISGs), inflammatory mediators, and chemokines. Interestingly, GO molecular function enrichment analysis also indicated significant enrichment of the STING-related innate immune response pathway in RT-treated ABCC10-knockout cells (Fig. 4c). To validate these results, RT-qPCR was performed, which revealed a marked increase in molecules related to the STING pathway at the mRNA level in RT-treated ABCC10-knockout cells; these included ISG15, IRF7, and MX1 (Supplementary Fig. 5a–c). In addition, key downstream modulators of the pathway, including phosphorylated TBK1, phosphorylated IRF3, and ISG15, were notably upregulated in the RT-treated groups (Fig. 4d and Supplementary Fig. 5d–g). Given the intrinsically low STING expression and phosphorylation levels in Patu8988T cells, we generated a STING-overexpressing Patu8988T cell line (Patu8988T^oe-STING^). Conversely, ISG15 mRNA expression, along with levels of pTBK1, pIRF3, pSTING, and ISG15 protein, were significantly decreased in ABCC10-overexpressing Patu8988T^oe-STING^ cells (Fig. 4e and Supplementary Fig. 5h–j).

To further clarify the role of the STING pathway in ABCC10-mediated reduction of ROS levels and DNA damage, ABCC10-knockout or ABCC10-knockdown cells were pretreated with or without H151, a specific inhibitor of STING (Supplementary Fig. 5k). Interestingly, H151 pretreatment significantly reduced intracellular ROS levels and γ-H2A.X protein levels in RT-treated ABCC10-knockout or ABCC10-knockdown cells (Fig. 4f, g and Supplementary Fig. 5l). In addition, cell viability and colony formation assays demonstrated that it had a protective effect, rescuing ABCC10-knockout cells from RT-induced cell death (Fig. 4h and Supplementary Fig. 5m). These findings indicate that the STING pathway functions downstream of ABCC10 in modulating RTR and is responsible for the accumulation of intracellular ROS and DNA damage.

### ABCC10 activates the STING pathway by exporting cGAMP in an ATP-dependent manner

**Fig. 5:**
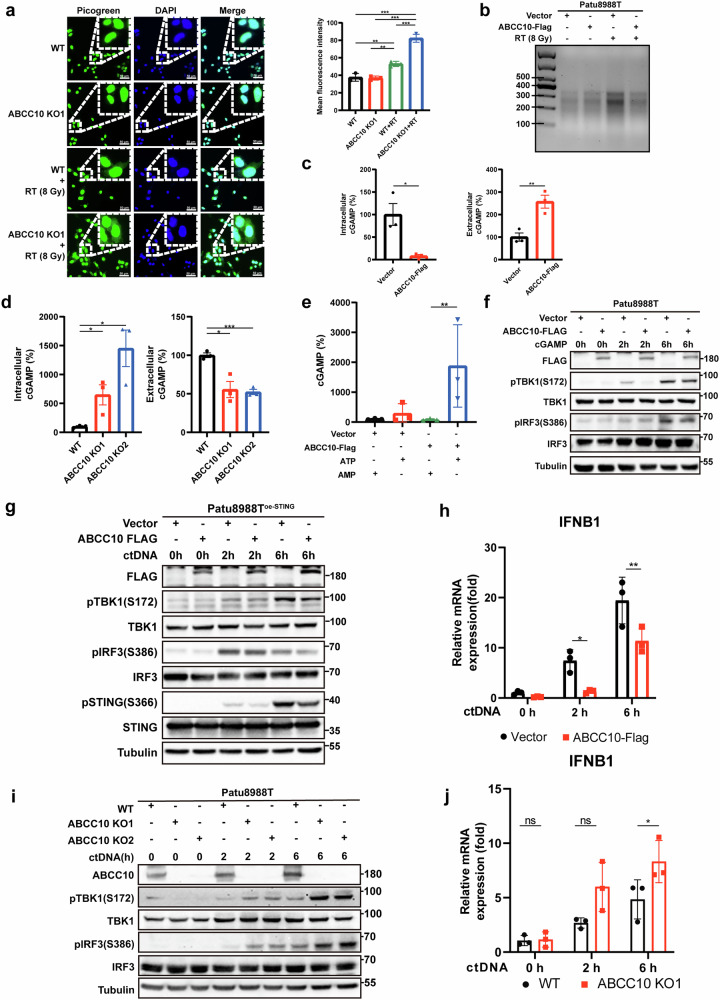
ABCC10-mediated cGAMP export limits activation of the STING pathway. **a** DNA was detected using PicoGreen dye in WT and ABCC10 KO1 cells. The nucleus was stained with DAPI dye. Fluorescence intensity of PicoGreen was calculated using ImageJ from three different areas, measuring the signal from PicoGreen in non-nuclear regions. **b** Cytoplasmic DNA from vector and ABCC10-overexpressing Patu8988T cell lysates was separated on an agarose gel. **c** Vector and ABCC10-overexpressing Patu8988T cells were treated with radiation (8 Gy) and measured for cGAMP in cell lysates and supernatants using an ELISA kit 8 h later. **d** WT and ABCC10-knockout Patu8988T cells were treated with radiation (8 Gy) and then measured for cGAMP in cell lysates and supernatants using an ELISA kit 8 h later. **e** 20-min vesicle transport assays using 293 T cell-derived vesicles expressing human ABCC10 or control vesicles with cGAMP (5 μM) in the presence of ATP or AMP. **f** Western blot analysis of vector and ABCC10-overexpressing cells treated with 2′3′-cGAMP after the indicated time points. **g** Western blot analysis of FLAG, ABCC10, pTBK1, TBK1, pIRF3, IRF3, pSTING, and STING expression in vector and ABCC10-overexpressing Patu8988T STING-OE cells. **h** qPCR analysis of IFNB1 mRNA expression in vector and ABCC10-overexpressing Patu8988T cells transfected with ctDNA at the indicated time points. **i** Western blot analysis of ABCC10 WT and KO Patu8988T cells transfected with ctDNA at the indicated time points. **j** qPCR analysis of IFNB1 mRNA expression in WT and ABCC10 KO1 Patu8988T cells transfected with ctDNA at the indicated time points. Experiments were repeated three times, and the data are expressed as mean ± SEM (*p < 0.05, **p < 0.01, ***p < 0.001, ****p < 0.0001).

As a classical cytoplasmic DNA sensor, cGAS contributes to the synthesis of cGAMP, which binds to the STING dimer, driving STING translocation from the endoplasmic reticulum to the Golgi apparatus and triggering the activation of the STING pathway. We hypothesized that ABCC10 activates the STING pathway by regulating cytoplasmic DNA levels and enhancing cGAS biosynthesis. PicoGreen staining revealed higher fluorescence intensity around the nucleus in RT-treated ABCC10-knockout cancer cells compared to other groups, indicating elevated cytoplasmic DNA levels (Fig. 5a). By contrast, ABCC10-overexpressing cancer cells displayed lower green fluorescence around the nucleus compared to empty vector cells (Supplementary Fig. 6a). In addition, nuclear/cytoplasmic fractionation of cancer cell lysates showed a significant reduction in cytoplasmic DNA content in RT-treated ABCC10-overexpressing cells (Fig. 5b). These results demonstrate that ABCC10 regulates cytoplasmic DNA levels during irradiation therapy. Interestingly, cGAS protein levels were mildly decreased in ABCC10-knockout cells but notably increased in ABCC10-overexpressing cells (Supplementary Fig. 6b–d).

Intrigued by the discrepancy between cGAS expression and STING pathway activation, we hypothesized that ABCC10 regulates the pathway by modulating intracellular cGAMP levels rather than directly altering cGAS expression. Given that ABCC10 is a member of the ATP-binding cassette (ABC) transporter family, we speculated that it might function as an efflux pump for cGAMP. To test this, we quantified intracellular and extracellular cGAMP levels using a 2′3′-cGAMP-specific ELISA. In these assays, extracellular cGAMP was directly measured from cell culture supernatants, whereas intracellular cGAMP was extracted from cell lysates. We observed that RT-treated ABCC10-overexpressing cancer cells exhibited significantly higher extracellular and lower intracellular cGAMP levels (Fig. 5c), supporting the efflux hypothesis. In contrast, ABCC10-knockout cells showed reduced extracellular and elevated intracellular cGAMP levels (Fig. 5d).

To directly assess whether ABCC10 can mediate ATP-dependent cGAMP transport, we performed in vitro vesicle transport assays. In this experiment, membrane vesicles prepared from ABCC10-overexpressing or control 293 T cells were incubated with 2′3′-cGAMP in the presence or absence of ATP. The amount of cGAMP retained within vesicles was quantified by ELISA after extensive washing. These assays confirmed that ABCC10-overexpressing vesicles accumulated significantly more cGAMP in an ATP-dependent manner, indicating that ABCC10 is capable of directly transporting cGAMP (Fig. 5e).

To investigate whether cGAMP efflux underlies the ABCC10-mediated activation of the STING pathway, we conducted rescue experiments by either supplying ABCC10-overexpressing cancer cells with exogenous cGAMP or treating them with ctDNA to enhance endogenous cGAMP production. As expected, the downregulation of pSTING, pTBK1, and pIRF3 at the protein level, as well as IFNB1 mRNA expression in ABCC10-overexpressing cells, was significantly reversed upon supplementation with either exogenous or endogenous cGAMP (Fig. 5f–h and Supplementary Fig. 6e). Conversely, in ABCC10-knockout or ABCC10-knockdown cancer cells, the upregulation of pTBK1, pIRF3, and IFNB1 was further amplified in the presence of both exogenous and endogenous cGAMP (Fig. 5i, j and Supplementary Fig. 6f, g). In addition, under ctDNA transfection conditions, ABCC10-overexpressing cells exhibited increased cGAMP efflux, while ABCC10-knockout cells showed intracellular cGAMP accumulation (Supplementary Fig. 6h, i).

**Fig. 6:**
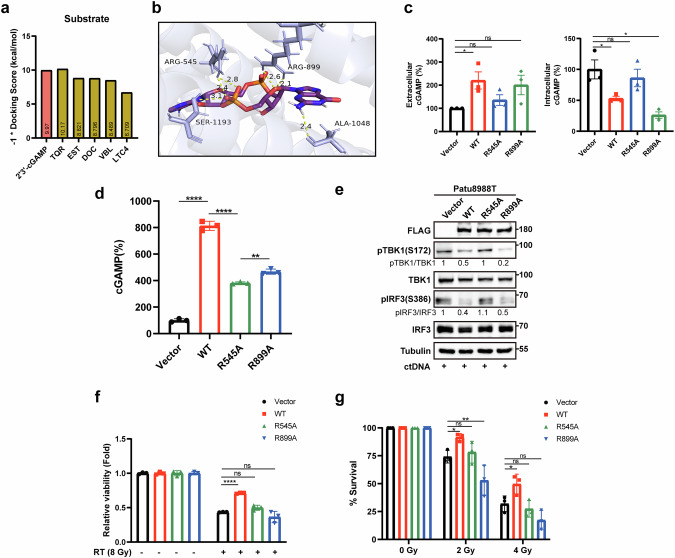
ABCC10 binds to cGAMP through the R545 site. **a** Docking scores × (-1) of ABCC10 substrates. **b** 2′3′-cGAMP docked with the ABCC10-binding pocket. **c** Patu8988T cells transfected with empty vector, wild-type (WT), R545A, or R899A mutant ABCC10-overexpression lentivirus were harvested after stimulation with ctDNA for 4 h. The 2′3′-cGAMP level in cell lysates and supernatants were measured using an ELISA kit. **d** 20-min vesicle transport assays using 293 T cell-derived vesicles expressing human WT ABCC10, R545A mutant, R899A mutant or control vesicles with cGAMP in the presence of ATP. **e** Western blot analysis of Patu8988T cells transduced with WT, R545A, R899A mutant ABCC10, or control (empty vector) stimulated with ctDNA for 4 h. **f** Cell viability of Patu8988T cells transfected with empty vector, WT, R545A, or R899A mutant ABCC10-overexpression lentivirus was assessed 72 h after RT. **g** Quantification of clonogenic survival analysis of vector, WT, R545A, or R899A mutant ABCC10-overexpressing Patu8988T cells treated with RT. Experiments were repeated three times, and the data are expressed as mean ± SEM (*p < 0.05, **p < 0.01, ***p < 0.001, ****p < 0.0001).

To further elucidate the relationship between ABCC10 and cGAMP, we first performed molecular docking analysis. Compared to previously identified ABCC10 substrates (E217βG and LTC4) and inhibitors (tariquidar, docetaxel, and vinblastine), cGAMP exhibited a high docking score, indicating a strong affinity for ABCC10 (Fig. 6a). According to the docking model, the R545 and R899 residues formed two hydrogen bonds with cGAMP, identifying them as key binding sites (Fig. 6b). Next, we generated two mutant cell lines (Patu8988T^R545A^ and Patu8988T^R899A^) and treated them with ctDNA to enhance endogenous cGAMP levels. Interestingly, while the elevated extracellular cGAMP levels observed in parental cells were reversed in Patu8988T^R545A^ cells, this reversal was not seen in Patu8988T^R899A^ cells (Fig. 6c). To directly assess the transport function of ABCC10 and its mutants, we performed a vesicle transport assay using inside-out membrane vesicles expressing wildtype ABCC10, R545A mutant, R899A mutant or empty vector. Vesicles were incubated with cGAMP in the presence of ATP, and the transported cGAMP was quantified using a sensitive ELISA assay. Consistent with the docking and cell-based findings, the R545A mutation significantly impaired cGAMP transport, while the R899A mutation had only a modest effect (Fig. 6d). In line with these observations, the suppression of pTBK1 and pIRF3, as well as the enhanced cell viability and clonogenic capacity in parental cells, were abrogated in Patu8988T^R545A^ cells, but not in Patu8988T^R899A^ cells (Fig. 6e–g). These findings indicate that cGAMP is pumped out in an ATP-dependent manner via its interaction with the R545 site of ABCC10.

### ABCC10 inhibitor exhibits robust synergistic effects with RT

**Fig. 7:**
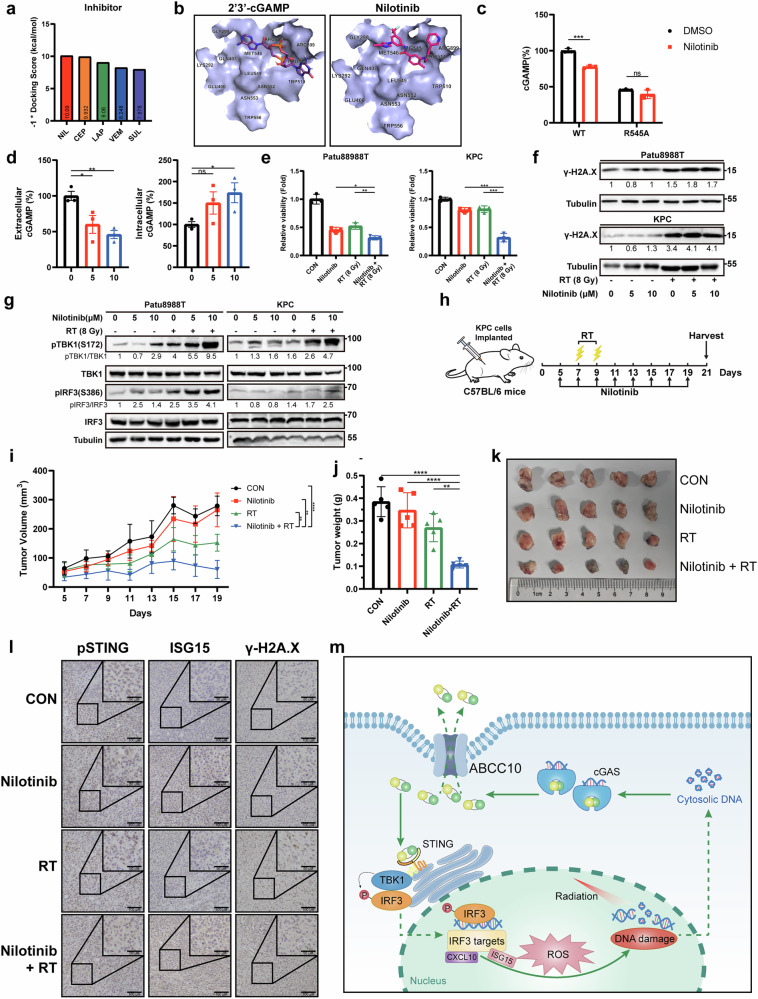
The ABCC10 inhibitor nilotinib enhances the therapeutic efficacy of radiotherapy both in vitro and in vivo. **a** Docking scores × (-1) of ABCC10 inhibitors. **b** Nilotinib docked with the ABCC10-binding pocket. **c** 20-min vesicle transport assays using 293 T cell-derived vesicles expressing human WT ABCC10 and R545A mutant with cGAMP and nilotinib in the presence of ATP. **d** ELISA was performed to detect intracellular and extracellular cGAMP content in Patu8988T cells treated with nilotinib stimulated with ctDNA for 4 h. **e** Cell viability was assessed using CCK-8 in Patu8988T and KPC mouse cells treated with nilotinib at 72 h after RT. **f** Western blot analysis of γ-H2A.X in Patu8988T and KPC mouse cells pretreated with nilotinib at 6 h after RT. **g** Western blot analysis of pTBK1, TBK1, pIRF3, and IRF3 protein levels in Patu8988T and KPC cells pretreated with nilotinib at 24 h after RT. **h** Schematic diagram of in vivo tumor growth and fractionated treatment protocol with radiation. Tumor growth curves (**i**) and tumor weights (**j**) for tumors generated from KPC mouse cells implanted subcutaneously in C57BL/6 mice with the indicated treatments. **k** Representative photographs of isolated tumor tissues following the indicated treatments. **l** Immunohistochemical analysis of pSTING, ISG15, and γ-H2A.X protein levels in harvested tumor tissues. Experiments were repeated three times, and the data are expressed as mean ± SEM (*p < 0.05, **p < 0.01, ***p < 0.001, ****p < 0.0001). Data were analyzed using Student’s t-test or two-way analysis of variance. **m** A schematic model showing the role of ABCC10 in radioresistance. ABCC10 blocks the activation of the STING pathway by causing cGAMP efflux after RT. Moreover, nilotinib can overcome radioresistance by inhibiting ABCC10 activity, thereby activating the STING pathway and inducing DNA damage.

To validate our in vitro findings, we first identified a potential ABCC10 inhibitor using docking analysis and found nilotinib to have the highest docking score (Fig. 7a). As an FDA-approved drug, nilotinib specifically competes with cGAMP to occupy the binding pocket in ABCC10 (Fig. 7b and Supplementary Fig. 7a). To provide direct functional evidence of the interaction between nilotinib and ABCC10, we conducted vesicular transport assays using membrane vesicles prepared from cells overexpressing either wild-type ABCC10 or the R545A mutant. Nilotinib significantly inhibited cGAMP uptake into vesicles containing wild-type ABCC10, whereas this inhibitory effect was largely abolished in vesicles harboring the R545A mutation (Fig. 7c). These results indicate that nilotinib suppresses ABCC10-mediated cGAMP transport in an R545-dependent manner, supporting a direct and functionally relevant interaction between nilotinib and the cGAMP-binding site of ABCC10. Nilotinib-treated cancer cells exhibited decreased extracellular cGAMP levels and increased protein expression levels of γ-H2A.X, pTBK1, and pIRF3, along with reduced cell viability in the presence of RT, suggesting that nilotinib may have a synergistic effect with RT (Fig. 7d–g). To determine whether this radiosensitizing effect of nilotinib is ABCC10-dependent, we performed combination treatment experiments in ABCC10 knockout (KO) cells. In contrast to wild-type cells, nilotinib failed to enhance the radiosensitivity of ABCC10 KO cells (Supplementary Fig. 7b, c), indicating that the synergistic effect of nilotinib with RT requires the presence of functional ABCC10.

C57BL/6 mice bearing KPC subcutaneous tumors were established and randomly divided into the following treatment groups (n = 5 mice per group): DMSO (control), nilotinib, RT, and nilotinib + RT (Fig. 7h). Compared to controls, all treatment groups exhibited inhibited tumor growth, with the most pronounced and durable effect observed in the nilotinib + RT group, indicating a synergistic effect of nilotinib with RT in vivo (Fig. 7i–k). Furthermore, immunohistochemistry results demonstrated enhanced expression levels of pSTING, pIRF3, and ISG15 in the nilotinib + RT group (Fig. 7l and Supplementary Fig. 7d).

The activation of STING and its downstream molecular events is critical for regulating anti-tumor immunity [22, 23]. We also assessed the infiltration and function of CD8^+^ T cells within the tumor tissue using flow cytometry analysis of tumors harvested from three mice per group. Nilotinib + RT treatment significantly enhanced the proportion of infiltrating CD8^+^ T cells, particularly TNFα^+^ and IFNγ^+^ CD8^+^ T cells (Supplementary Fig. 7d–g). This may explain the durable tumor inhibition observed with nilotinib + RT treatment. These results further substantiate the evidence that nilotinib can synergistically enhance the therapeutic efficacy of RT by activating the STING pathway and promoting CD8^+^ T cell immune infiltration.

## Discussion

The DNA damage response and immune escape significantly limit the effectiveness of RT. In this study, using CRISPR-Cas9 metabolic screening, we identified ABCC10 as a critical guardian against DNA damage and a driver of immune escape, promoting cancer cell resistance to RT (Fig. 7m). Interestingly, we found that ABCC10 can efflux cell-intrinsic cGAMP, which subsequently suppresses the STING pathway and alleviates intercellular ROS accumulation and DNA damage. Importantly, the combination of the ABCC10 inhibitor nilotinib and RT exhibited a synergistic effect in inhibiting tumor growth and eliciting anti-tumor immunity. Thus, ABCC10 has the potential to serve as both a therapeutic target for overcoming RTR and a sensitive biomarker for evaluating RT responses.

As the principal intracellular DNA sensor, the cGAS-STING pathway has emerged as a key element of innate immunity and a promising therapeutic target [24]. In the context of cancer, upon engagement by DNA, the cGAS-STING pathway can impact virtually all aspects of tumorigenesis, from cancer cell transformation to metastasis [24]. In the precancerous stage of tumors, transformed cells exhibit characteristics such as telomere shortening, chromosomal abnormalities, and replicative crisis. Under such cellular stress, the cGAS-STING pathway dictates transformed cell death via autophagy, rather than eliciting canonical cytokine signaling [9]. As cancer cells evolve, cGAS–STING activity can induce either the type I IFN response or canonical NF-κB-dependent cytokine signaling. Based on its function, cGAS–STING can both directly prevent cancer cell proliferation and promote immune cell-mediated cell death [25]. Once the tumor is established, persistently activated cGAS-STING pathways have distinct tumor-promoting effects. Chronic activation of the cGAS-STING pathway in cancer cells enhances non-canonical NF-κB signaling, which facilitates metastasis. In addition, this signaling inhibits dendritic cell function, further contributing to an immunosuppressive tumor microenvironment. Thus, the cGAS-STING pathway displays a dual role, either promoting or inhibiting tumors, depending on the timing and magnitude of STING stimulation. Our results revealed that ABCC10 suppresses the STING-TBK1-IRF3 pathway, thereby facilitating cancer cell resistance to RT both in vitro and in vivo, consistent with previous publications regarding STING activation and RT efficiency [11].

cGAMP is a key element of the cGAS-STING pathway, exerting its effects both as an intracellular second messenger and through export to neighboring cells within the tumor microenvironment. It can move between cells via gap junctions or be packaged into enveloped viral particles [26, 27]. Identifying cGAMP transporters has become a hot topic in cell biology. SLC19A1 was the first identified cGAMP conduit in human monocytic cell lines, contributing to cGAMP import and subsequent STING activation. Later, SLC46A2, P2X7, and LRRC8A were gradually recognized as potential importers. Maltbaek et al. [28] recently identified ABCC1 as the first cGAMP exporter, which limits cell-intrinsic STING activation in autoimmune diseases. In line with this, our study is the first to discover that ABCC10 functions as a cGAMP exporter in an ATP-dependent manner in cancer cells. These findings not only broaden our understanding of how cGAMP exits cells but also suggest that existing cGAMP exporters may share structural similarities.

As a superfamily of membrane proteins, ABCC transporters are well-known for their ability to transport a wide variety of exogenous and endogenous substances across cell membranes. In the context of cancer, ABCC transporters have been implicated in mediating the efflux of chemotherapeutic compounds (or their metabolites) from malignant cells. With deeper investigation, it has become evident that ABCC transporters are also involved in the movement of intrinsic biomaterials within cancer cells. Due to variation in tissue distribution and membrane localization, each ABCC transporter exhibits distinct pharmacological and physiological functions [29, 30]. Although some ABCC members may share substrate specificity, there are usually significant differences in transport kinetics for common substrates. Using high-throughput CRISPR-Cas9 metabolic screening, we precisely identified ABCC10 as a mediator of RTR, rather than other ABCC members. ABCC10 expression is widely detected in various cancers, including breast, lung, ovarian, and pancreatic cancers [31, 32]. However, we were unable to confirm this observation in the clinical samples we collected. This may be attributed to the limited sample size and the absence of critical data regarding timing and magnitude of expression, which warrants further investigation in future studies.

In summary, we identified ABCC10 as a critical molecular site for cGAMP export in the context of cancer RT. Our findings demonstrate that ABCC10-mediated cGAMP export functions as a negative regulator of cell-intrinsic STING signaling, thereby reducing intracellular ROS accumulation, mitigating DNA damage, and promoting cancer cell resistance to RT. We propose that highly specific ABCC10 inhibitors could offer an effective therapeutic strategy for overcoming RTR in a significant subset of patients. Furthermore, the expression levels of ABCC10 may serve as a potential biomarker for predicting the responsiveness of cancer patients to RT.

## Materials and methods

### Cell culture

The Patu8988T cell line was obtained from Procell Life Science & Technology Company (China), the MCF-7 cell line was obtained from Shanghai WHELAB Bioscience Limited (China), while the BxPC3 and Calu-1 cell line were purchased from Fuheng Biology Company (China). The KPC cell line was acquired from the Shanghai Model Organisms Center. All cell lines were cultured in DMEM supplemented with 10% FBS, 100 U/mL penicillin, and 0.1 mg/mL streptomycin. Cells were maintained in a humidified atmosphere of 5% CO_2_ at 37 °C. Unless otherwise specified, the cell culture medium was changed every 72 h, and cells were passaged using 0.05% trypsin/EDTA.

### Radioresistant cell lines

The radioresistant Patu8988T cell line was developed from the parental Patu8988T cell line by repeated exposure to 2 Gy fractions of X-ray irradiation, reaching a cumulative dose of 40 Gy.

### Reagents and antibodies

Nilotinib (T1524), H-151 (T5674), and Lapatinib (T0078) were purchased from TOPSCIENCE. Acetylcysteine (NAC, HY-B0215) was obtained from MedChemExpress, and 2′3′-cGAMP (B8362) was acquired from APExBIO.

Primary antibodies used for Western blotting were as follows: anti-β-tubulin (Engibody, AT0011, 1:10,000), anti-Histone H3 (Proteintech, 17168-1-AP, 1:5000), anti-ABCC10 (Proteintech, 30697-1-AP, 1:1000), anti-phospho-IRF3 (Ser386) (Abclonal, AP0995, 1:1000), anti-IRF3 (Proteintech, 11312–1-AP, 1:10,000), anti-phospho-STING (Ser366) (Abclonal, AP1369, 1:1000), anti-STING (Proteintech, 19851–1-AP, 1:2000), anti-phospho-TBK1 (CST, #5483, 1:1,000), anti-TBK1 (CST, #38066, 1:1,000), anti-γ-H2A.X (Abcam, ab243906, 1:1000), anti-CGAS (Proteintech, 26416–1-AP, 1:2,000), and anti-FLAG (Proteintech, 20543–1-AP, 1:20,000). Secondary antibodies (anti-rabbit or anti-mouse) were purchased from Abclonal.

The antibodies used for flow cytometry were anti-CD45 FITC (BioLegend, #103108, 1:200), anti-CD8a PE (BioLegend, #100708, 1:200), anti-IFNγ Percp/Cy5.5 (BioLegend, #505822, 1:200), and anti-TNFα BV650 (BioLegend, #506333, 1:200).

### Metabolic CRISPR/Cas9 sgRNA library screen

The Human CRISPR Metabolic Gene Knockout library, a gift from David Sabatini (Addgene #110066), was amplified according to the supplier’s protocol. The metabolic sgRNA library was packaged using the PMD2.G envelope plasmid and the psPAX2 lentiviral packaging plasmid. Patu8988T/BxPC3 cells were infected with lentivirus at a multiplicity of infection of 0.3, maintaining 500× coverage of the library.

Twenty-four hours after lentiviral infection, cells were cultured in normal DMEM medium for 2 days, followed by selection with 2 µg/mL puromycin for an additional 2 days. After a 48 h recovery post-selection, cells were irradiated every 2 days with a single 4 Gy dose using a linear accelerator (TrueBeam-STX, Varian, USA) for 6 days. Unirradiated cells were continuously cultured for 6 days as a control group.

Genomic DNA isolation was performed using the QIAGEN Plasmid Plus Maxi Kit (#12963), and gRNA sequences were amplified using a two-step PCR process, ensuring ~500× coverage of the metabolic library. Sequencing was performed using an Illumina Novaseq 6000 to generate 150 bp paired-end reads. Raw counts were assessed using MAGeCK (version 0.5.9.5) with the command “mageck test -k RTvsCtrl.count.txt -t rt -c ctrl --norm-method control --control-sgrna control-sgrna.txt.” The results of the library screening, analyzed using MAGeCK, are presented in Supplementary Tables 1 and 2.

### Data analysis

To analyze ABCC10 expression in tumor and normal tissues, the combined transcript expression dataset from GTEx and TCGA was obtained via UCSC Xena (http://xena.ucsc.edu/). To analyze ABCC10 expression in parallel and radioresistant MCF-7 cells, the expression profile from the GSE120798 dataset was used.

For survival analysis of patients undergoing RT, clinical data from BRCA, HNSC, and PAAD patients were obtained from UCSC Xena (http://xena.ucsc.edu/). Patients with recorded RT information were selected and stratified into high and low expression groups based on the median ABCC10 expression level. Overall survival curves were generated using the “survival” and “survminer” R packages.

### Cell viability measurement

Cell viability was assessed using the Cell Counting Kit-8 (CCK-8, APExBIO) following the manufacturer’s instructions. Briefly, 1000 cells were seeded into each well of a 96-well plate and treated as specified. The optical density at 450 nm (OD450) was measured 2 h after the CCK-8 reagent was added. Cell viability was calculated according to the manufacturer’s guidelines.

### Colony-forming assay

In the cell colony formation assay, 1000 cells were seeded into six-well plates and cultured for 10–14 days. Afterward, the cells were fixed with 4% paraformaldehyde and stained using crystal violet staining solution. Colonies were quantified using ImageJ software.

### Constructs and generation of, knockout, knockdown, or overexpression cell lines

To establish ABCC10-knockout cell lines, we employed a dual sgRNA targeting strategy to enhance gene-editing efficiency. The gRNAs targeting distinct regions of ABCC10 were designed using the CRISPOR website tool (http://crispor.tefor.net/). Oligonucleotides containing dual sgRNAs, along with an additional gRNA scaffold, were synthesized and cloned into the LentiCRISPR V2 puro vector at the BsmBI site.

To establish DOX-inducible lentiviral knockdown cell lines, ABCC10 shRNA was inserted into the Tet-pLKO-Puro vector. For cell experiments, the culture medium was replaced with DOX (1 μg/mL) 24 h prior to experimentation to induce ABCC10 knockdown.

To generate dCas9-mediated ABCC10-expressing cell lines, cells were infected with lentivirus containing the Lenti-dCAS-VP64-Blast vector. After 2 weeks of selection with blasticidin, the cells were transduced with lentivirus containing pCRISPRia-v2 cloned with targeting gRNAs and selected with puromycin for an additional week. The sequences of the gRNA and shRNA are listed in Supplementary Table 5.

Wild-type (WT) and mutant human ABCC10 constructs were generated using a human ABCC10 cDNA clone (Sino Biological HG19038-UT) as a template. Then the PCR products were cloned into the pCDH-CMV-puro vector. For the rescue experiments, to reintroduce ABCC10 expression in the knockout cell line, the cDNA of ABCC10 was cloned into the pLV-CMV-BLAST vector, which confers blasticidin resistance. For the STING overexpression construct, the human STING cDNA clone was synthesized and subsequently cloned into the pLV-CMV-BLAST vector.

### Lentivirus production

For virus production, we transfected 293 T cells with the constructed plasmids, along with packaging plasmids (psPAX2 and pMD2G). After 48 and 72 h, the supernatant was collected, centrifuged, and filtered through 0.45 µm filters.

### Western blot

Whole-cell lysates were prepared in RIPA buffer (50 mM Tris-HCl, pH 8.0, 150 mM NaCl, 0.1% SDS, 0.5% Na-Deoxycholate, 1% Triton X-100) supplemented with the cOmplete protease inhibitor cocktail (Roche). Protein quantification was performed using the BCA protein assay (Vazyme). Considering the tendency of ABCC10 protein to aggregate upon boiling, we added nucleases to the samples to reduce viscosity, followed by a 15 min incubation at 37 °C. All other samples were boiled for 10 min. Then the proteins were separated on a 10% or 12% Bis-Tris SDS-PAGE gel and transferred to a PVDF membrane (Millipore). Quantification of the relative expression level of target protein was normalized to Tubulin unless otherwise indicated. The uncropped original western blots are provided in the “Supplemental Material” file.

### Vesicle transport assays

To examine whether ABCC10 mediates ATP-dependent cGAMP transport, we conducted vesicle transport assays using membrane vesicles derived from 293T cells overexpressing wildtype ABCC10, R545A mutant, R899A mutant or empty vector. Briefly, cells in the logarithmic growth phase were harvested and washed with cold PBS containing 1% aprotinin. After centrifugation at 4000 × *g* for 5 min, the cells were suspended in HM buffer (0.25 M sucrose, 1 mM EDTA, 20 mM HEPES, pH 7.4) and homogenized gently in a glass homogenizer. Then the lysate was subjected to centrifugation at 4,000 × g to remove cell nuclei and unlysed cells. The supernatant was carefully applied onto a sucrose cushion (10 mM Tris–HCl, 35% sucrose, 1 mM EDTA, pH 7.4) and centrifuged for 30 min at 16,000 × *g* at 4 °C. The interface was collected and further centrifuged at 100,000 × g for 45 min at 4 °C. The precipitate was resuspended in incubation buffer (10 mM Tris-HCl, 10 mM MgCl2, 250 mM sucrose, pH 7.0) by repeated passage through a 27-gauge needle.

Vesicle transport assays were conducted to determine whether 2′3′-cGAMP serves as a direct substrate for ABCC10. Before the experiment began, all reagents were dissolved in the incubation buffer and pre-warmed at 37 °C for 3 min. To examine the competitive interaction between nilotinib and cGAMP for ABCC10 binding, nilotinib (10 μM) and 2′3′-cGAMP (5 μM) were co-incubated with membrane vesicles derived from either wild-type or R545A mutant ABCC10. The reaction was initiated by the addition of ATP (5 mM) or AMP (5 mM), and the resulting 500 μL mixtures were incubated at 37 °C for 20 min. The reactions were halted by placing the tubes on ice and adding 3 mL pre-chilled wash buffer (10 mM Tris-HCl, 100 mM NaCl, 0.25 M sucrose, pH 7.4) to each tube. Then the samples were rapidly passed through 0.22 μm PVDF filters (Millipore) that had been presoaked in the wash buffer. The filters were washed three times with 3 mL wash buffer and eluted using 1 mL RIPA buffer. Samples were subsequently measured using the 2′3′-cGAMP ELISA Kit (Cayman #501700). The measured cGAMP levels were normalized to protein concentration, which was determined using the bicinchoninic acid (BCA) protein assay.

### Quantitative RT-PCR

Total RNA from cancer cells or tissues was extracted, and the RNA was reverse transcribed into cDNA using an RNA extraction kit (Accurate Biology, AG21023) and a reverse transcription kit (Vazyme, R323–01) according to the manufacturer’s guidelines. Following this, triplicate SYBR Green-based real-time PCR analyses were conducted using SYBR Green master mix (TransGen, AQ131) on an Applied Biosystems QuantStudio™ 3 PCR machine (Thermo Fisher Scientific). The threshold cycle (Ct) values of each gene were standardized based on the expression levels of ACTB. The sequences of primers, synthesized, and purified by GenScript, are listed in Supplementary Table 6.

### ROS detection

Cells were seeded in a 12-well plate and irradiated as indicated. The culture medium was removed, and the cells were washed twice with PBS. Then they were incubated with DCFH-DA mixed in DMEM at 37 °C for 30 min. After washing the adherent cells with PBS three times, the stained cells were harvested using trypsinization and resuspended in PBS. ROS levels were assessed by flow cytometry (FITC 488 nm) with 1 × 10^4^ cells. The gating strategy for DCFH-DA ROS experiments is shown in Supplementary Fig. 8a.

### RNA sequencing

Total RNA was extracted from the cells of both the control and treatment groups using Trizol. The integrity and concentration of the RNA were assessed utilizing the RNA Nano 6000 Assay Kit on the Bioanalyzer 2100 system (Agilent Technologies, CA, USA). A total of 2 µg RNA per sample was used as input material for RNA sample preparations. Sequencing libraries were synthesized using the NEBNext Ultra RNA Library Prep Kit for Illumina (#E7530L, NEB, USA) according to the manufacturer’s guidelines, incorporating index codes to assign sequences to each respective sample. Following library quality control, the clustering of the index-coded samples was performed, and the libraries were sequenced on an Illumina platform, producing 150 bp paired-end reads.

RNA-seq reads were quality-filtered and trimmed using trim_galore_v0.6.7. The clean reads from the samples were mapped to the human genome reference (GRCh38 release 109), retrieved from the Ensembl database (http://www.ensembl.org), using HISAT2_v2.2.1 software. Then the reads were counted with featureCounts_v2.0.1. Differential gene expression analysis was performed following the limma_voom pipeline (limma_v3.54.0), and the results are shown in Supplementary Tables 3 and 4. Gene Ontology (GO) enrichment analysis was conducted using Metascape (https://metascape.org).

### Cytoplasmic DNA detection

Cells were seeded in a 12-well plate prior to irradiation. After irradiation for 6 h, the cells were washed three times with cold PBS and fixed in 4% paraformaldehyde for 30 min. Following three additional PBS washes, cells underwent a blocking step with 1% BSA in PBS for 1 h, followed by a 1 h staining period with Pico488 dsDNA quantification reagent and a 15 min staining with DAPI. Each well was photographed with a Leica confocal microscope after being washed three times with PBS. To quantify cytoplasmic DNA, fluorescence intensity analysis was performed using ImageJ, measuring the signal from PicoGreen in non-nuclear regions (outside of DAPI staining).

For DNA agarose gel analysis, cell samples underwent binary fission, with one portion dedicated to cytoplasmic DNA extraction and the other to total protein extraction. Nuclear and cytoplasmic components were isolated using a nuclear and cytoplasmic extraction kit. Then the cytoplasmic DNA was extracted using a DNA extraction kit (Accurate Biology, AG21010). The remaining half of the cell samples was used for total protein extraction with RIPA buffer. The loading amount of cytoplasmic DNA for agarose gel analysis was adjusted based on the protein concentration quantified using the BCA method.

### cGAMP measurements

Intracellular and extracellular 2′3′-cGAMP levels were quantified using the 2′3′-cGAMP ELISA Kit (Cayman #501700) according to the manufacturer’s instructions. This assay is based on competition between native 2′3′-cGAMP in samples and a fixed amount of 2′3′-cGAMP conjugated to horseradish peroxidase (2′3′-cGAMP-HRP Tracer) for binding to a limited amount of polyclonal anti-2′3′-cGAMP antibody. The resulting signal inversely correlates with the amount of free 2′3′-cGAMP present in the sample.

Indicated cells were seeded in 12-well plates 24 h prior to stimulation. For extracellular cGAMP measurements, cell supernatants (500 μL) were collected 8 h after radiation or 4 h after ctDNA stimulation and directly subjected to ELISA. For intracellular cGAMP measurements, cells were washed three times with pre-chilled PBS and then lysed in 500 μL RIPA buffer (150 mM NaCl, 1% Triton X-100, 0.5% sodium deoxycholate, 0.1% SDS, 50 mM Tris pH 8.0) for 15 min on ice. Insoluble components in the lysates were removed through centrifugation. Then the relative cGAMP measurements were quantified using the 2′3′-cGAMP ELISA Kit.

### Xenograft experiments

Animal studies were approved by the Committee on the Use of Live Animals for Teaching and Research of the Jiangsu University. Male NXG immunodeficient mice and C57BL/6 mice (purchased from Cavens Laboratory Animal) were subjected to daily health monitoring throughout the experiment. They were housed in a controlled environment with a regular 12 h light–dark cycle, provided with a standard diet, and maintained in a pathogen-free barrier facility. Different investigators were responsible for tumor induction, drug delivery, and assessing outcomes such as tumor size and body weight, thereby ensuring blinding.

For xenografts of Patu8988T cells, 1 × 10^7^ DOX-induced ABCC10-knockdown cells were implanted subcutaneously into the right dorsal flanks of NXG immunodeficient mice. Mice were randomized into the specified groups (n = 5 per group). Mice in the ABCC10-knockdown group were provided with doxycycline (2 mg/mL) in their drinking water starting on day 5 after tumor implantation. Radiation (4 Gy) was administered to mice on days 9 and 11. Tumor volume and body weight were monitored every 2 days.

To investigate the synergistic therapeutic effect of nilotinib in sensitizing RT, 2 × 10^6^ KPC (Pdx1-cre/LSL-Kras G12D/P53[R172H]) cells were implanted subcutaneously into the right dorsal flanks of C57BL/6 mice (n = 5 per group). Nilotinib was administered every 2 days via intraperitoneal injection to the mice 5 days after tumor implantation. Tumor volume and body weight were monitored every 2 days. All experimental procedures were approved by the Committee on the Use of Live Animals for Teaching and Research of Jiangsu University. For assessing CD8+ infiltration and function, tumors were harvested from mice sacrificed after the indicated treatment (n = 3 per group). The tumor lesions were manually dissociated using surgical scissors and a syringe piston, and single-cell suspensions were prepared using a 70 µm cell strainer. For surface marker analysis, single cells were stained with anti-CD45 and anti-CD8a antibodies for 20 min at 4 °C. Following surface staining, the cells were fixed, permeabilized, and stained with anti-TNFα and anti-IFNγ antibodies for 30 min at 4 °C. Data were acquired by flow cytometry and analyzed using FlowJo or Cytexpert software.

### Statistical analysis

Across all experiments, biological replicate data (n) are reported as mean values with either standard deviation (SD) or standard error of the mean (SEM), as indicated. Statistical analysis was conducted using GraphPad Prism 8.3 software, as detailed in the Results section. *P*-values ≤ 0.05 were considered indicative of statistical significance. All tests were two-tailed unless otherwise specified.

## Supplementary information

Supplementary Figures

Supplementary Tables

Uncropped WB data

## Data Availability

Data is provided within the manuscript or supplementary information files. The remaining data are available from the authors upon request.

## References

[1] Delaney G, Jacob S, Featherstone C, Barton M. The role of radiotherapy in cancer treatment. Cancer. 2005;104:1129–37. 16080176 10.1002/cncr.21324

[2] Begg AC, Stewart FA, Vens C. Strategies to improve radiotherapy with targeted drugs. Nat Rev Cancer. 2011;11:239–53. 21430696 10.1038/nrc3007

[3] An L, Li M, Jia Q. Mechanisms of radiotherapy resistance and radiosensitization strategies for esophageal squamous cell carcinoma. Mol Cancer. 2023;22:140. 37598158 10.1186/s12943-023-01839-2 PMC10439611

[4] Barker HE, Paget JTE, Khan AA, Harrington KJ. The tumour microenvironment after radiotherapy: mechanisms of resistance and recurrence. Nat Rev Cancer. 2015;15:409–25. 26105538 10.1038/nrc3958 PMC4896389

[5] Hanahan D, Weinberg RA. Hallmarks of cancer: the next generation. Cell. 2011;144:646–74. 21376230 10.1016/j.cell.2011.02.013

[6] Morandi A, Indraccolo S. Linking metabolic reprogramming to therapy resistance in cancer. Biochim Biophys Acta Rev Cancer. 2017;1868:1–6. 28065746 10.1016/j.bbcan.2016.12.004

[7] Schieber M, Chandel NS. ROS function in redox signaling and oxidative stress. Curr Biol. 2014;24:R453–R462. 24845678 10.1016/j.cub.2014.03.034 PMC4055301

[8] McBrayer SK, Mayers JR, DiNatale GJ, Shi DD, Khanal J, Chakraborty AA, et al. Transaminase inhibition by 2-hydroxyglutarate impairs glutamate biosynthesis and redox homeostasis in glioma. Cell. 2018;175:101–116.e25. 30220459 10.1016/j.cell.2018.08.038 PMC6219629

[9] Nassour J, Radford R, Correia A, Fusté JM, Schoell B, Jauch A, et al. Autophagic cell death restricts chromosomal instability during replicative crisis. Nature. 2019;565:659–663. 30675059 10.1038/s41586-019-0885-0 PMC6557118

[10] Ranoa DRE, Widau RC, Mallon S, Parekh AD, Nicolae CM, Huang X, et al. STING promotes homeostasis via regulation of cell proliferation and chromosomal stability. Cancer Res. 2019;79:1465–79. 30482772 10.1158/0008-5472.CAN-18-1972 PMC6445702

[11] Hou Y, Liang H, Rao E, Zheng W, Huang X, Deng L, et al. Non-canonical NF-κB antagonizes STING sensor-mediated DNA sensing in radiotherapy. Immunity. 2018;49:490–503.e4. 30170810 10.1016/j.immuni.2018.07.008 PMC6775781

[12] Sun L, Wu J, Du F, Chen X, Chen ZJ. Cyclic GMP-AMP synthase is a cytosolic DNA sensor that activates the type I interferon pathway. Science. 2013;339:786–91. 23258413 10.1126/science.1232458 PMC3863629

[13] Chen Q, Boire A, Jin X, Valiente M, Er EE, Lopez-Soto A, et al. Carcinoma–astrocyte gap junctions promote brain metastasis by cGAMP transfer. Nature. 2016;533:493–8. 27225120 10.1038/nature18268 PMC5021195

[14] Yan N, Regalado-Magdos AD, Stiggelbout B, Lee-Kirsch MA, Lieberman J. The cytosolic exonuclease TREX1 inhibits the innate immune response to human immunodeficiency virus type 1. Nat Immunol. 2010;11:1005–13. 20871604 10.1038/ni.1941 PMC2958248

[15] Carozza JA, Böhnert V, Nguyen KC, Skariah G, Shaw KE, Brown JA, et al. Extracellular cGAMP is a cancer-cell-produced immunotransmitter involved in radiation-induced anticancer immunity. Nat Cancer. 2020;1:184–96. 33768207 10.1038/s43018-020-0028-4 PMC7990037

[16] Sundararaman SK, Barbie DA. Tumor cGAMP awakens the natural killers. Immunity. 2018;49:585–7. 30332624 10.1016/j.immuni.2018.10.001 PMC6390837

[17] Marcus A, Mao AJ, Lensink-Vasan M, Wang L, Vance RE, Raulet DH. Tumor-derived cGAMP triggers a STING-mediated interferon response in non-tumor cells to activate the NK cell response. Immunity. 2018;49:754–763.e4. 30332631 10.1016/j.immuni.2018.09.016 PMC6488306

[18] Seshacharyulu P, Baine MJ, Souchek JJ, Menning M, Kaur S, Yan Y, et al. Biological determinants of radioresistance and their remediation in pancreatic cancer. Biochim Biophys Acta. 2017;1868:69–92. 10.1016/j.bbcan.2017.02.003 PMC5548591 28249796

[19] Bassik MC, Kampmann M, Lebbink RJ, Wang S, Hein MY, Poser I, et al. A systematic mammalian genetic interaction map reveals pathways underlying ricin susceptibility. Cell. 2013;152:909–22. 23394947 10.1016/j.cell.2013.01.030 PMC3652613

[20] Zhou W, Yao Y, Scott AJ, Wilder-Romans K, Dresser JJ, Werner CK, et al. Purine metabolism regulates DNA repair and therapy resistance in glioblastoma. Nat Commun. 2020;11:3811. 32732914 10.1038/s41467-020-17512-x PMC7393131

[21] Kim TW, Hong D-W, Hong SH. CB13, a novel PPARγ ligand, overcomes radio-resistance via ROS generation and ER stress in human non-small cell lung cancer. Cell Death Dis. 2020;11:848. 33051435 10.1038/s41419-020-03065-w PMC7555888

[22] Zhang Y, Zhai Q, Feng X, Chen D, Lu Y, Hu J, et al. Cancer cell-intrinsic STING is associated with CD8 + T-cell infiltration and might serve as a potential immunotherapeutic target in hepatocellular carcinoma. Clin Transl Oncol Publ Fed Span Oncol Soc Natl Cancer Inst Mex. 2021;23:1314–24. 10.1007/s12094-020-02519-z 33502741

[23] Luo B, Zhang S, Yu X, Tan D, Wang Y, Wang M. Gasdermin E benefits CD8+T cell mediated anti-immunity through mitochondrial damage to activate cGAS-STING-interferonβ axis in colorectal cancer. Biomark Res. 2024;12:59. 38853246 10.1186/s40364-024-00606-9 PMC11163757

[24] Samson N, Ablasser A. The cGAS–STING pathway and cancer. Nat Cancer. 2022;3:1452–63. 36510011 10.1038/s43018-022-00468-w

[25] Decout A, Katz JD, Venkatraman S, Ablasser A. The cGAS-STING pathway as a therapeutic target in inflammatory diseases. Nat Rev Immunol. 2021;21:548–69. 33833439 10.1038/s41577-021-00524-z PMC8029610

[26] Ablasser A, Schmid-Burgk JL, Hemmerling I, Horvath GL, Schmidt T, Latz E, et al. Cell intrinsic immunity spreads to bystander cells via the intercellular transfer of cGAMP. Nature. 2013;503:530–4. 24077100 10.1038/nature12640 PMC4142317

[27] Gentili M, Kowal J, Tkach M, Satoh T, Lahaye X, Conrad C, et al. Transmission of innate immune signaling by packaging of cGAMP in viral particles. Science. 2015;349:1232–6. 26229115 10.1126/science.aab3628

[28] Maltbaek JH, Cambier S, Snyder JM, Stetson DB. ABCC1 transporter exports the immunostimulatory cyclic dinucleotide cGAMP. Immunity. 2022;55:1799–1812.e4. 36070769 10.1016/j.immuni.2022.08.006 PMC9561016

[29] Slot AJ, Molinski SV, Cole SPC. Mammalian multidrug-resistance proteins (MRPs). Essays Biochem. 2011;50:179–207. 21967058 10.1042/bse0500179

[30] Chen Z, Tiwari AK. Multidrug resistance proteins (MRPs/ABCCs) in cancer chemotherapy and genetic diseases. FEBS J. 2011;278:3226–45. 21740521 10.1111/j.1742-4658.2011.08235.x PMC3168698

[31] Kathawala RJ, Wang Y-J, Ashby CR, Chen Z-S. Recent advances regarding the role of ABC subfamily C member 10 (ABCC10) in the efflux of antitumor drugs. Chin J Cancer. 2014;33:223–30. 24103790 10.5732/cjc.013.10122 PMC4026542

[32] Wang J-Q, Cui Q, Lei Z-N, Teng Q-X, Ji N, Lin L, et al. Insights on the structure-function relationship of human multidrug resistance protein 7 (MRP7/ABCC10) from molecular dynamics simulations and docking studies. MedComm. 2021;2:221–35. 34766143 10.1002/mco2.65 PMC8491190

