# Supplementary material for: ABCC10-mediated cGAMP efflux drives cancer cell radiotherapy resistance
Source: Cell Death Differ. 2025 Aug 6;33(1):111–25. doi: 10.1038/s41418-025-01552-1 (PMC12811381; doi:10.1038/s41418-025-01552-1)
Supplement: Supplementary file 1 — Supplementary Figures [file 41418_2025_1552_MOESM1_ESM.docx]

Supplementary Information for:

**ABCC10-mediated cGAMP efflux drives cancer cell radiotherapy resistance**

Zhengyang Zhang^1#^, Jie Gao^1,2#^, Xiang Liao^1#^, Zining Zhang^1^, Xiongfeng Cao^1,2^, Yi Gong^1^, Wenlong Chen^1^, Lirong Zhang^1,2^, Hsiang-i Tsai^1,2*^, Dongqing Wang^1,2*^, Haitao Zhu^1,2*^

1. Institute of Medical Imaging and Artificial Intelligence, Jiangsu University, Zhenjiang, China, 212001
2. Department of medical imaging, The Affiliated Hospital of Jiangsu University, Zhenjiang, China, 212001


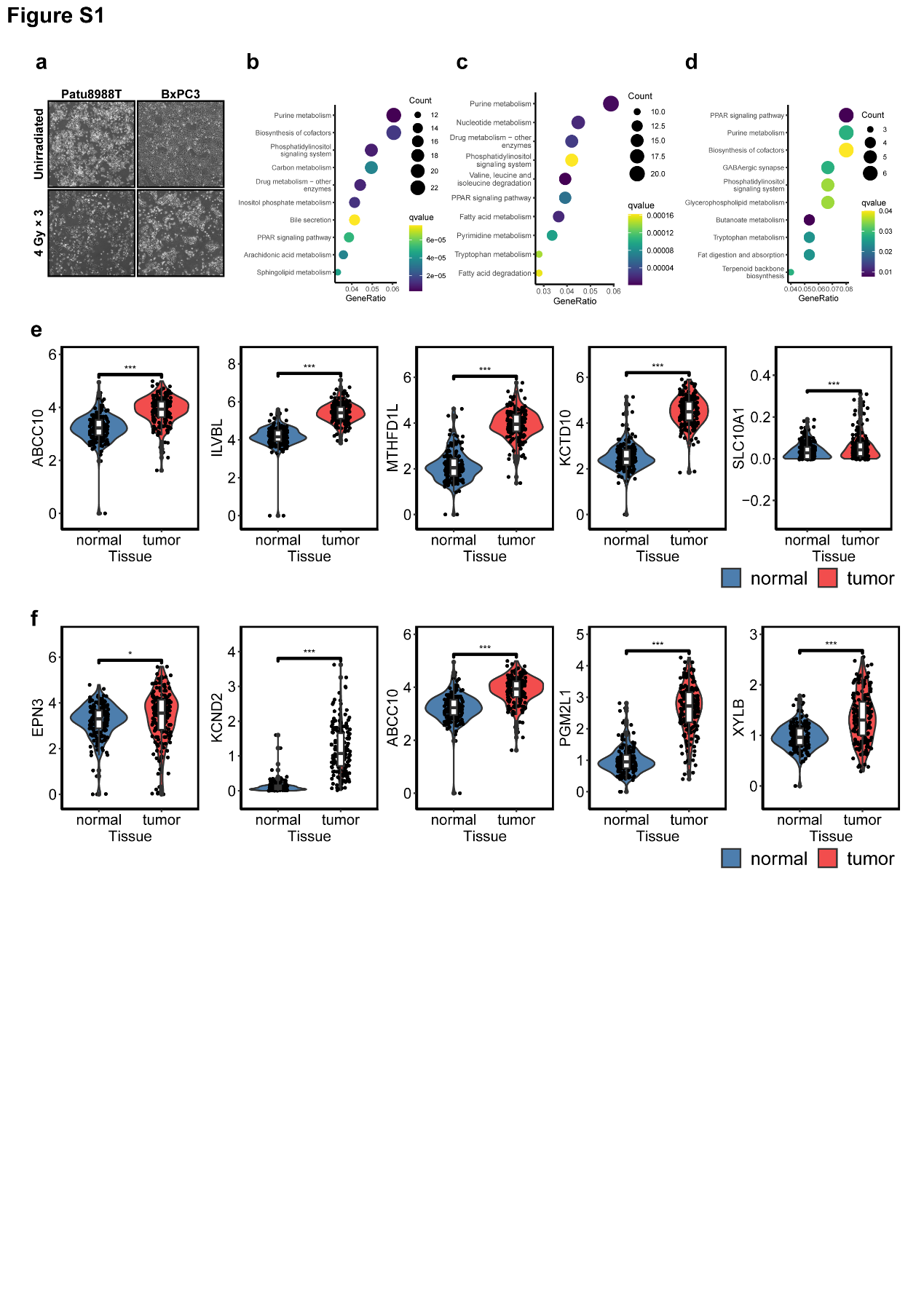


**Supplementary Figure 1. a** Images of cell density following 3 x 4 Gy radiotherapy treatment. **b, c** KEGG enrichment analysis of significant radioresistant gene in the screen of Patu8988T (**b**) and BxPC3 (**c**) cells. **d** KEGG enrichment analysis for the intersection of 99 negative regulatory genes. **e, f** TCGA analysis of top 5 negative regulatory genes mRNA expression in the screen of Patu8988T (**e**) and BxPC3 (**f**) cells.


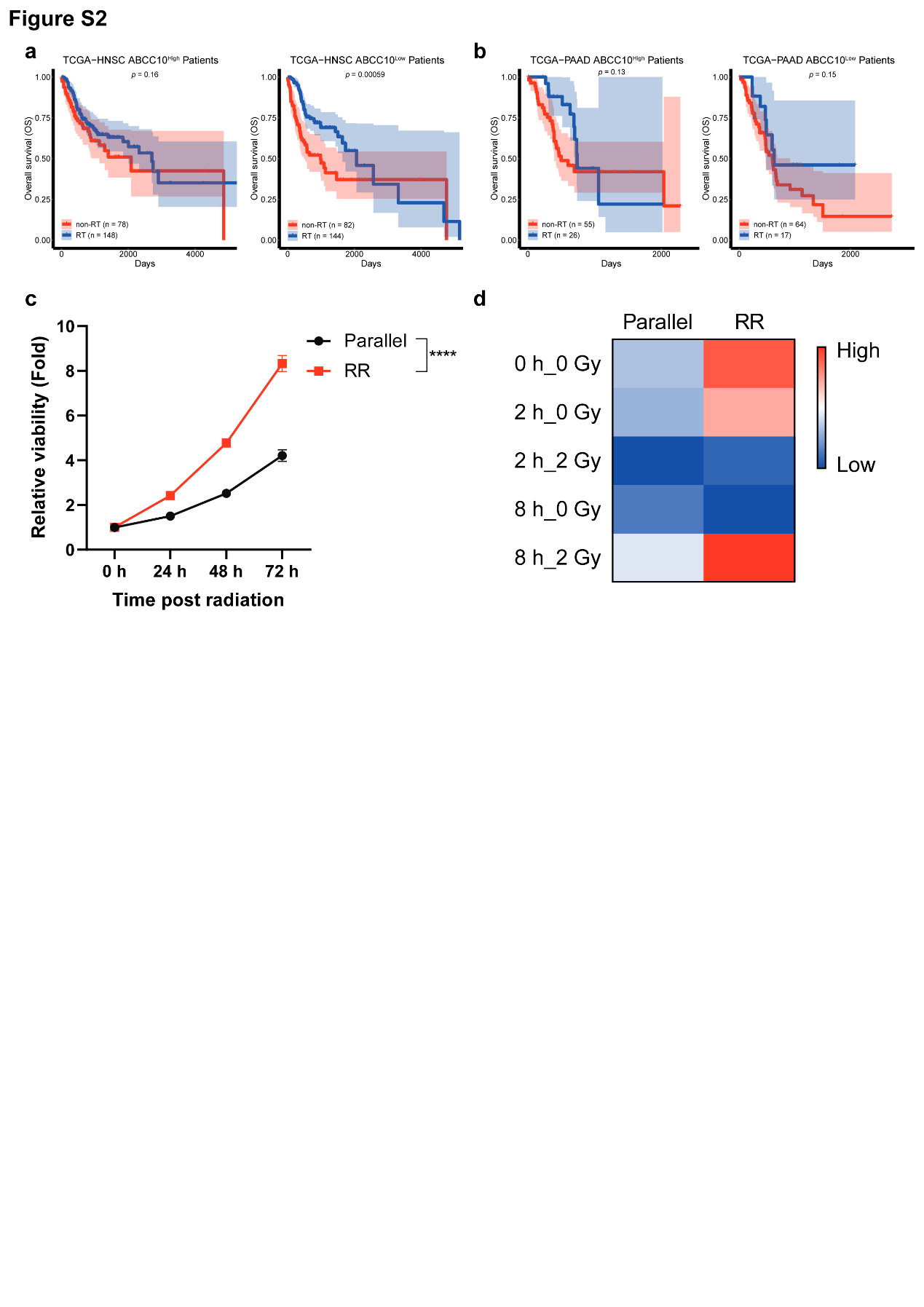


**Supplementary Figure 2. a, b** Survival analysis of HNSC (**a**) and PAAD (**b**) patients with high or low ABCC10 expression treated with or without radiotherapy. **c** Cell viability of parallel and radioresistant Patu8988T cells treated with RT at indicated time points. **d** Heatmap of ABCC10 expression in parallel and radioresistant MCF-7 cells treated with or without RT (GSE120798).


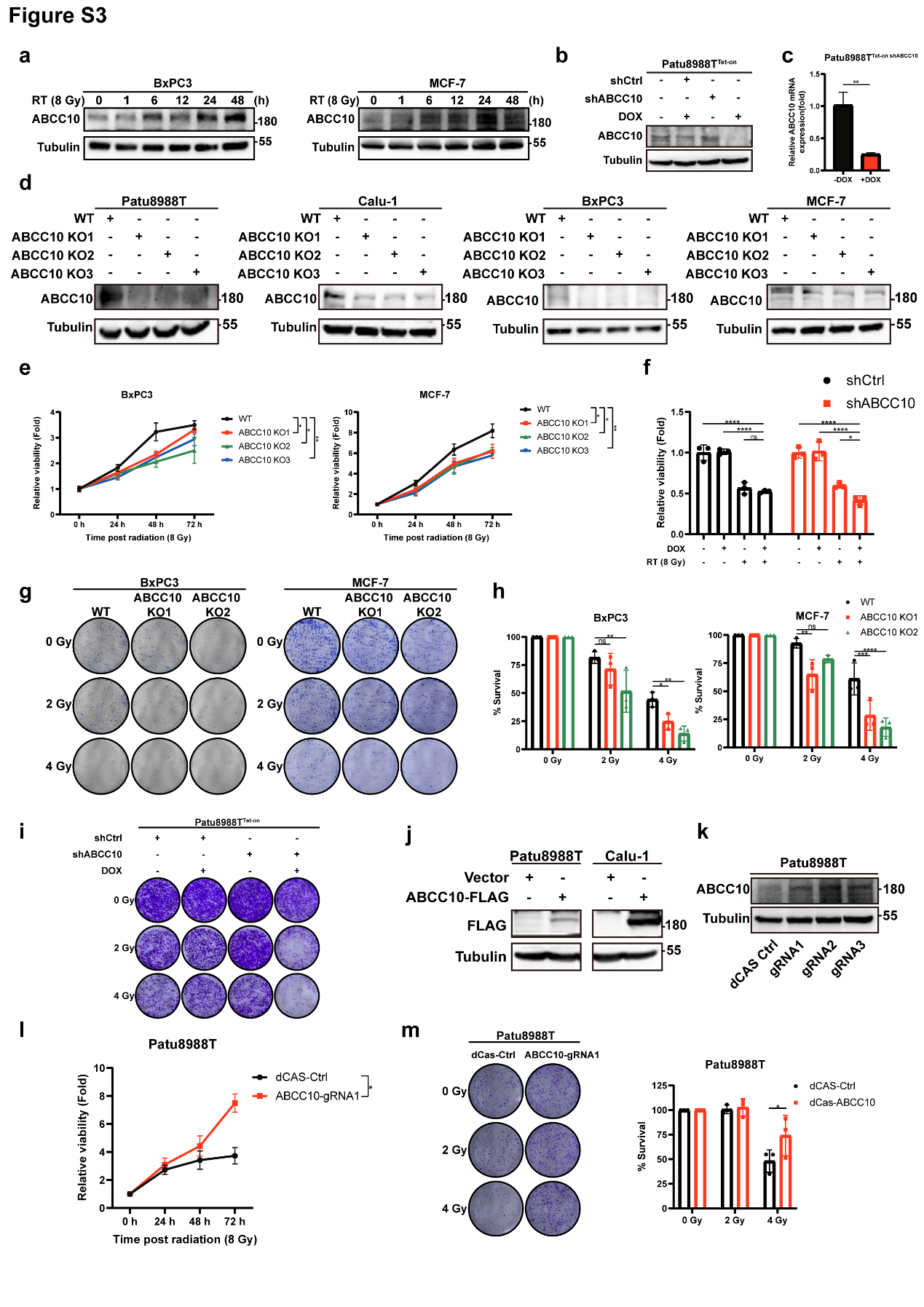


**Supplementary Figure 3. a** Immunoblot analysis of the protein expression levels of ABCC10 in BxPC3 and MCF7 cells treated with RT (8 Gy). **b, c** Protein expression levels (**b**) and mRNA expression levels (**c**) of ABCC10 in doxycycline-inducible shCtrl and shABCC10 Patu8988T cells treated with or without doxycycline. **d** Patu8988T, Calu-1, BxPC3 and MCF-7 cells were transduced with lentiCRISPR encoding ABCC10 or control gRNAs, selected for 3 days, and ABCC10 protein levels were assessed by immunoblot. **e** Cell viability in BxPC3 and MCF-7 ABCC10 knockout cells treated with RT (8 Gy). **f** Cell viability of doxycycline-inducible shCtrl and shABCC10 Patu8988T cells pre-treated with or without doxycycline at 72h after RT (8 Gy). **g, h** Representative images (**g**) and quantiﬁcation (**h**) of clonogenic survival analysis of BxPC3 and MCF-7 ABCC10 knockout cells treated with the indicated dose of ionizing radiation. **i** Representative images of clonogenic survival analysis of doxycycline-inducible shCtrl and shABCC10 Patu8988T cells treated with the indicated dose of ionizing radiation. **j** Western blot analysis of FLAG protein expression in the empty vector and ABCC10-FLAG overexpressing Patu8988T and Calu-1 cells. **k** Western blot analysis of ABCC10 expression in control and dCAS mediated ABCC10 transcriptional activated Patu8988T cells. **l** Cell viability in control and dCAS mediated ABCC10 transcriptional activated Patu8988T cells treated with RT. **m** Representative images and quantification of clonogenic survival analysis of the control and dCAS mediated ABCC10 transcriptional activated Patu8988T cells treated with the indicated dose of ionizing radiation.


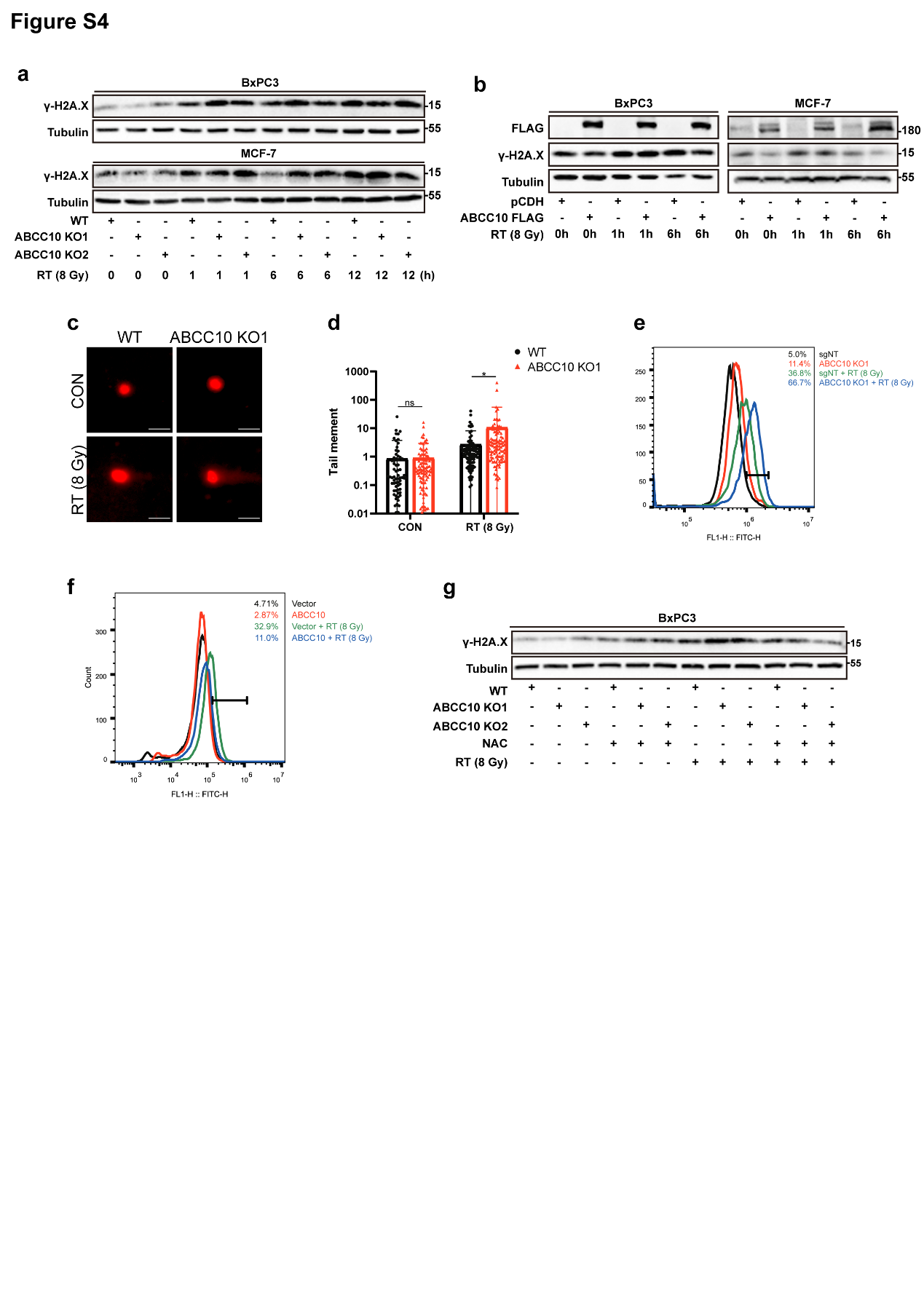


**Supplementary Figure 4. a** Immunoblot analysis of the protein expression levels of γ-H2A.X in BxPC3 and MCF-7 ABCC10 knockout cells at the indicated times after RT. **b** Immunoblot analysis of the protein expression levels of γ-H2A.X in BxPC3 and MCF-7 ABCC10 overexpressing cells at the indicated times after RT. **c, d** Representative images (**c**) and quantification (**d**) of neutral comet assay foci performed in wild-type (WT) and ABCC10-knockout (KO) Patu8988T cells treated with RT. Scale bars, 50 μm. **e** Representative curves of ﬂow cytometry-based analysis of ROS (measured by CM-H2DCFDA) levels in Patu8988T WT and ABCC10 KO1 cells at 24 hours treated with RT. **f** Representative curves of ﬂow cytometry-based analysis of ROS (measured by CM-H2DCFDA) levels in Patu8988T vector and ABCC10 overexpressing cells at 24 hours treated with RT. **g** Immunoblot analysis of the protein expression levels of γ-H2A.X in WT and ABCC10 KO BxPC3 cells pretreated with or without NAC (1 mM) at 6 h after RT.


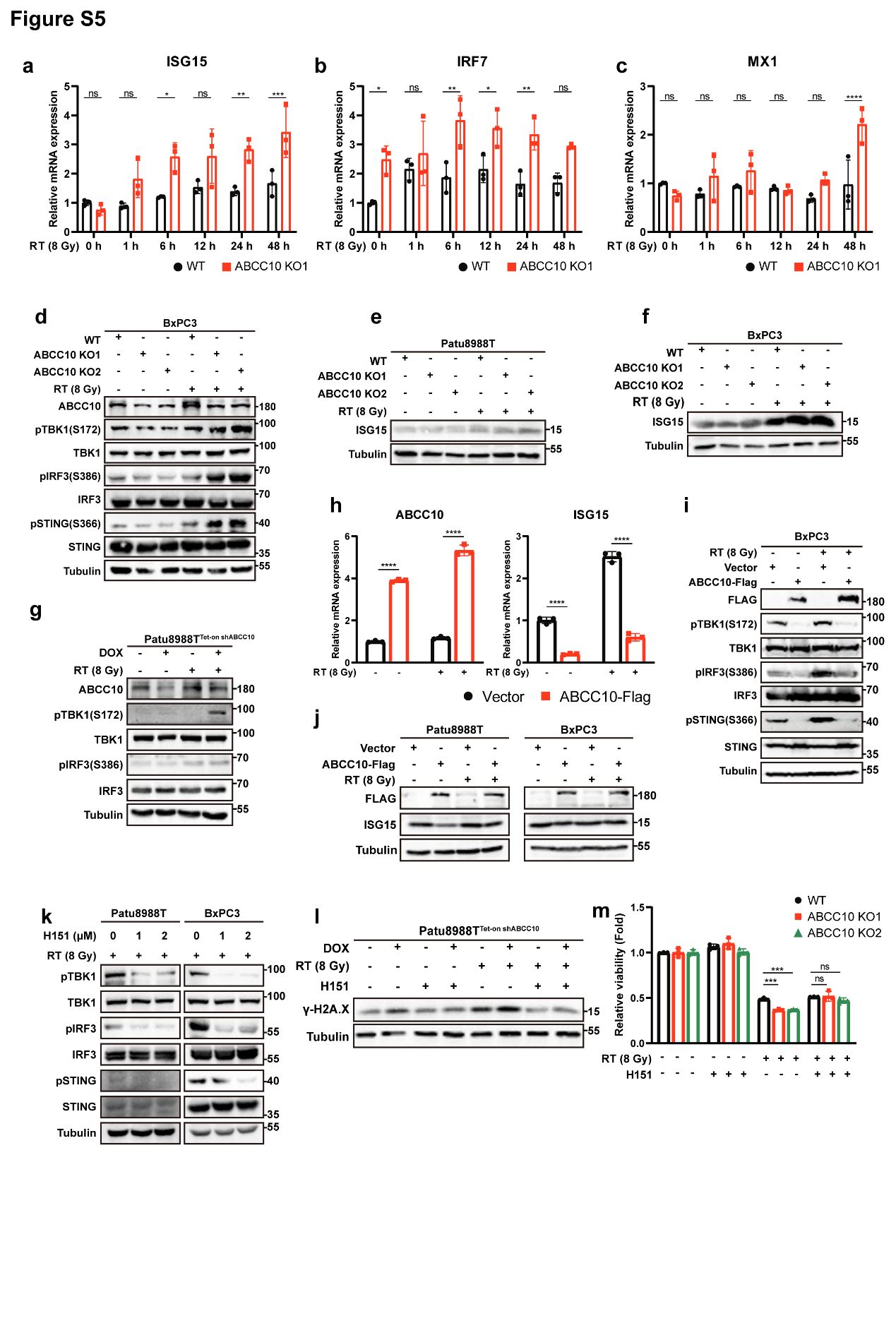


**Supplementary Figure 5. a­–c** qPCR analysis of ISG15 (**a**), IRF7 (**b**), and MX1 (**c**) mRNA levels in Patu8988T cells after RT (8 Gy) at the indicated time points. **d** Western blot analysis of ABCC10, pTBK1, TBK1, pIRF3, IRF3, pSTING and STING protein levels in ABCC10 WT and KO BxPC3 cells. **e, f** Western blot of ISG15 protein level in WT and ABCC10 KO Patu8988T (**e**) and BxPC3 (**f**) cells at 24h after RT (8 Gy). **g** Western blot analysis of ABCC10, pTBK1, TBK1, pIRF3, IRF3, and cGAS protein levels in doxycycline-inducible knockdown Patu8988T cells treated with RT (8 Gy). **h** qPCR analysis of ISG15 and ABCC10 mRNA levels in the empty vector and ABCC10-FLAG overexpressing Patu8988T cells at 24h after RT (8 Gy). **i** Western blot analysis of FLAG, pTBK1, TBK1, pIRF3, IRF3 pSTING and STING protein levels in vector and ABCC10 overexpressing BxPC3 cells. **j** Western blot of ISG15 and FLAG protein level in the empty vector and ABCC10-FLAG overexpressing cells at 24h after RT (8 Gy). **k** Western blot analysis of pTBK1, TBK1, pIRF3, IRF3 pSTING and STING protein levels in Patu8988T and BxPC3 cells treated with H151 or not. **l** Western blot analysis of γ-H2A.X protein levels in doxycycline-inducible knockdown Patu8988T cells pretreated with doxycycline or H151 at 6 hours treated with RT. **m** Cell viability of Patu8988T WT and ABCC10 knockout cells pretreated with or without H151 (1 μM) at 72 h after RT.


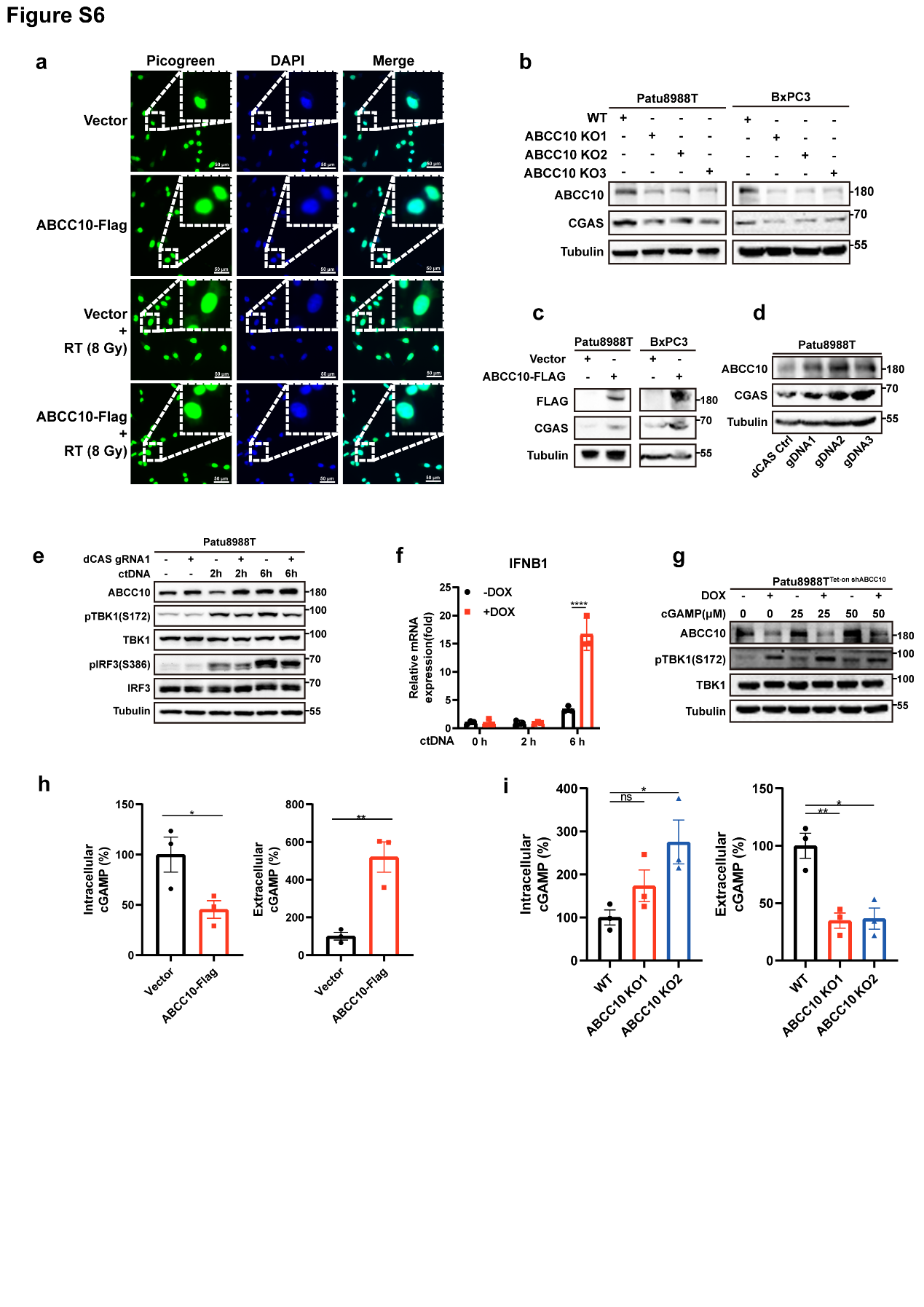


**Supplementary Figure 6. a** DNA was detected using the PicoGreen dye in the empty vector and ABCC10 overexpressing cells. Nuclear was stained with DAPI dye. **b** Western blot analysis of cGAS protein level in WT and ABCC10 knockout cells. **c** Western blot analysis of cGAS protein level in the empty vector and ABCC10 expressing cells. **d** Western blot analysis of cGAS protein level in the control and dCAS mediated ABCC10 transcriptional activated Patu8988T cells. **e** Western blot analysis of ABCC10, pTBK1, TBK1, pIRF3, IRF3 and cGAS expression in the control and dCAS mediated ABCC10 transcriptional activated Patu8988T cells transfected with ctDNA at indicated time points. **f** qPCR analysis of IFNB1 mRNA expression in doxycycline-inducible knockdown Patu8988T cells pretreated with doxycycline transfected with ctDNA at indicated time points. **g** Western blot analysis of ABCC10, pTBK1 and TBK1 expression in doxycycline-inducible knockdown Patu8988T cells treated with cGAMP for 4 hours. **h** Vector and ABCC10 overexpressing Patu8988T cells were treated with ctDNA and then 4 h later cGAMP was measured in cell lysates and supernatants using ELISA kit. **i** WT and ABCC10 KO Patu8988T cells were treated with ctDNA and then 4 h later cGAMP was measured in cell lysates and supernatants using ELISA kit.


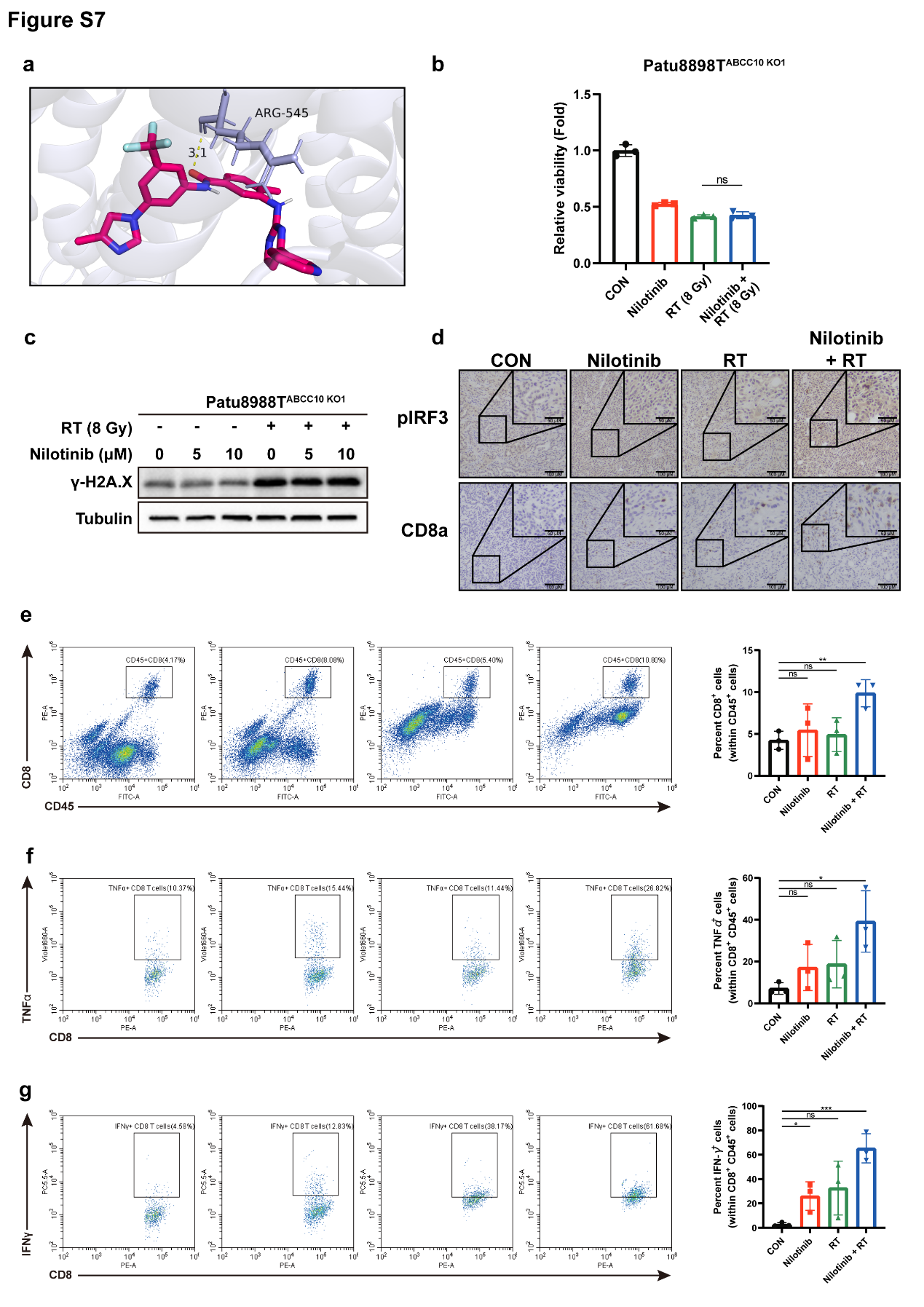


**Supplementary Figure 7. a** Nilotinib docked with the ABCC10 binding pocket. **b** Cell viability was assessed in ABCC10 KO1 Patu8988T cells treated with nilotinib at 72 h after RT. **c** Western blot analysis of γ-H2A.X in ABCC10 KO1 Patu8988T cells pretreated with nilotinib for 24 hours at 6 h after RT. **d** IHC analysis of pIRF3 and CD8a protein levels in harvested tumor tissues. **e** Analysis of percentage of CD8^+^ T cells infiltration in harvested tumor tissues by flow cytometry. **f, g** Analysis of percentage of TNFα^+^ and IFNγ^+^ cells within CD8^+^ T cells in harvested tumor tissues by flow cytometry**.**


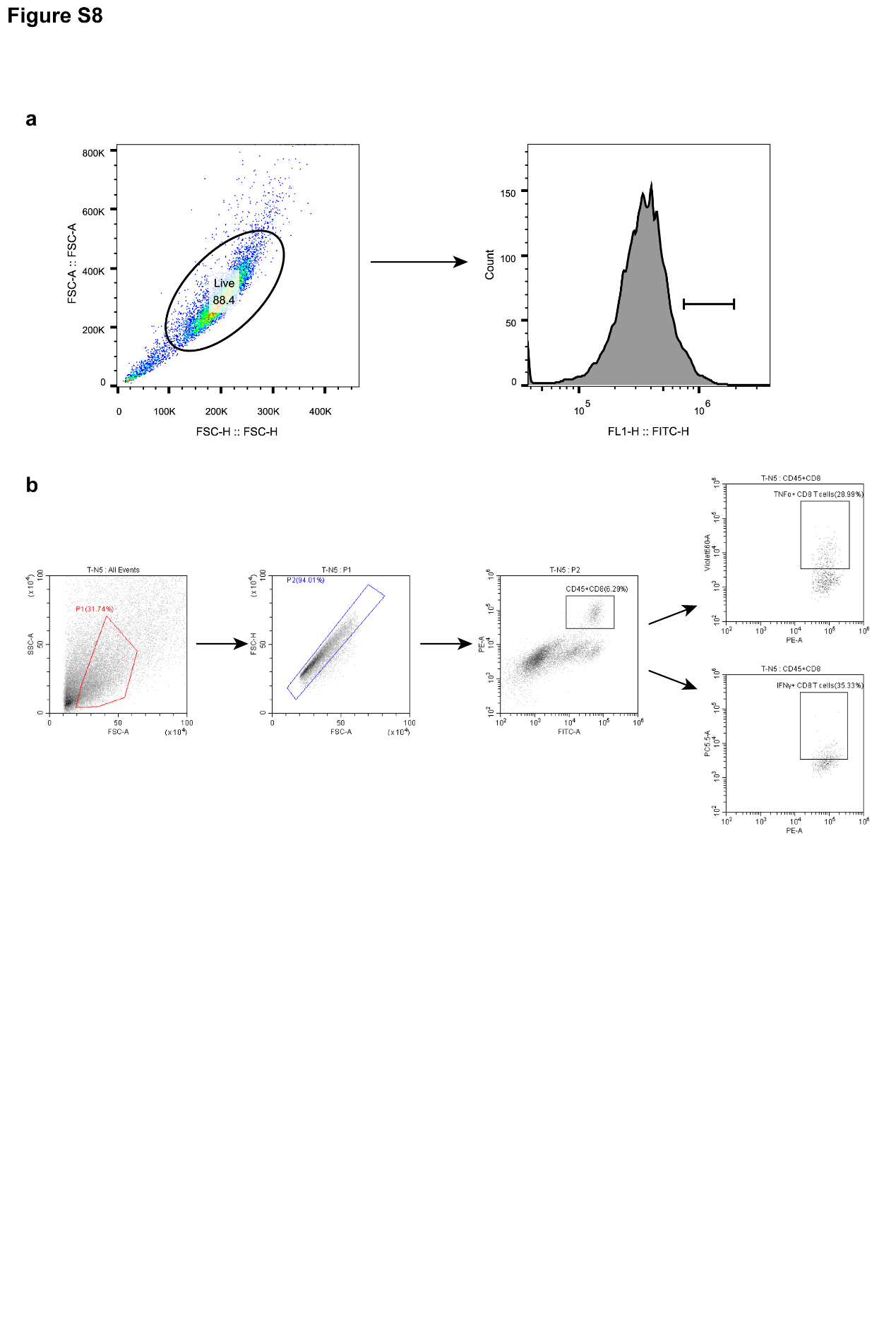


**Supplementary Figure 8. a** Gating strategy for DCFH-DA ROS experiments. **b** Gating strategy for analysis of CD8^+^ T cells infiltration and functions experiments.
